# TRP Channels as Molecular Targets to Relieve Endocrine-Related Diseases

**DOI:** 10.3389/fmolb.2022.895814

**Published:** 2022-04-28

**Authors:** Yusheng Liu, Yihan Lyu, Hongmei Wang

**Affiliations:** Department of Pharmacology, School of Medicine, Southeast University, Nanjing, China

**Keywords:** TRP, transient receptor potential channels, gland, endocrine-related diseases, calcium channels

## Abstract

Transient receptor potential (TRP) channels are polymodal channels capable of sensing environmental stimuli, which are widely expressed on the plasma membrane of cells and play an essential role in the physiological or pathological processes of cells as sensors. TRPs often form functional homo- or heterotetramers that act as cation channels to flow Na^+^ and Ca^2+^, change membrane potential and [Ca^2+^]_i_ (cytosolic [Ca^2+^]), and change protein expression levels, channel attributes, and regulatory factors. Under normal circumstances, various TRP channels respond to intracellular and extracellular stimuli such as temperature, pH, osmotic pressure, chemicals, cytokines, and cell damage and depletion of Ca^2+^ reserves. As cation transport channels and physical and chemical stimulation receptors, TRPs play an important role in regulating secretion, interfering with cell proliferation, and affecting neural activity in these glands and their adenocarcinoma cells. Many studies have proved that TRPs are widely distributed in the pancreas, adrenal gland, and other glands. This article reviews the specific regulatory mechanisms of various TRP channels in some common glands (pancreas, salivary gland, lacrimal gland, adrenal gland, mammary gland, gallbladder, and sweat gland).

## Introduction

Endocrine gland-related diseases, though rare, remain a significant threat. Take the islets as an example, pancreatitis can contribute to diabetes and pancreatic cancer, and the existing treatment options, such as radiotherapy and surgery, are ineffective (Kleeff et al., 2017; Hart and Conwell, 2020). In addition, the cases of pancreatic ductal adenocarcinoma (PDAC) are on the rise year by year, and the fatality rate is very high because most cases are found in the late stage. For non-early-stage patients, existing systemic therapy, radiotherapy, and cytotoxic therapy are not complete cures (Park et al., 2021). In this regard, regulation of TRPs can help regulate the microenvironment of pancreatic cells and improve the possibility of a specific therapy.

On the other hand, standard treatments for salivary and lacrimal gland-associated Sjögren’s syndrome (pSS) include anti-inflammatory drugs, steroids, hormones, immunosuppressants, and biotherapy (Ramos-Casals et al., 2020). Primary adrenal insufficiency or hyperplasia is also often treated with hormone replacement therapy to mimic normal secretion. Nevertheless, the death rate among primary adrenal insufficiency patients continues increasing (El-Maouche et al., 2017; Barthel et al., 2019). For hidradenitis suppurativa (acne inversa), surgical, laser, and antibiotic treatments are often inadequate, new drug targets are needed, and patients are often associated with a range of endocrine diseases such as obesity, diabetes, and metabolic syndrome (Saunte and Jemec, 2017; Sabat et al., 2020).

Moreover, studies have proved that COVID-19 is associated with secretory gland diseases such as diabetes, adrenal insufficiency, and mumps (De-Madaria and Capurso, 2021; Fisher et al., 2021; Zhou et al., 2021). Patients with diabetes, obesity, and primary adrenal insufficiency infected with COVID-19 will have more severe damage to the endocrine glands, resulting in a poor prognosis and the possibility of chronic inflammation, significantly increasing hospitalization, severe illness, and mortality rates (Marazuela et al., 2020; Zhou et al., 2021). In contrast, COVID-19 can induce diabetic onset in patients with hyperglycemia and induce secondary adrenal insufficiency. In addition, the hormone therapy previously used in patients with endocrine diseases should be changed accordingly after COVID-19 infection (Lisco et al., 2021). Here, the TRP channels are also noteworthy as potential drug targets for endocrine glands.

## Development and Classification of TRP Channels

TRP channels have been gradually discovered and studied in the past few decades. The TRP channel was initially named after the photo-transducted channels in *Drosophila melanogaster*, which is blind to constant bright light (Cosens and Manning, 1969; Hardie, 2011; Samanta et al., 2018). Various TRPs discovered later were classified according to differences in amino acid sequence and topological structure, including seven families in total: TRPC (canonical), TRPV (vanilloid), TRPM (melastatin), TRPA (ankyrin), TRPP (polycystic), TRPML (mucolipin), and TRPN (*Drosophila* NOMPC) (Hardie, 2011; Li, 2017; Samanta et al., 2018). And then there is TRPY, which was just discovered in yeast and named after it (Amini et al., 2021). Among them, TRPC, TRPV, TRPM, and TRPA are group1 of TRP channels, which have high similarities with *drosophila* TRP channels. TRPP and TRPML are group 2 of TRP channels, which have distal relevance to *drosophila* TRP channels. Group1 and 2 are different in the transmembrane domain (Li, 2017; Samanta et al., 2018).

Due to its homology with *drosophila* TRP sequence, TRPCs were first discovered and had seven members, divided into four subgroups: TRPC1, TRPC2 (a pseudogene in mammals), TRPC3\6\7, and TRPC4\5. TRPCs can be activated by gated receptors, storage operations, and mechanical stimuli to participate in cell regulation (Wes et al., 1995; Vazquez et al., 2004; Ong et al., 2016). TRPVs are named after the activation of TRPV1 by capsaicin in sensory neurons (Caterina et al., 1999). In addition, vanillin and vanillic acid in plants can also activate TRPVs (Li, 2017). TRPVs have six members, divided into two subgroups: TRPV1-4 and TRPV5\6. TRPV1-4 are thermosensitive nonselective nociceptors, which are related to neural plasticity (Satheesh et al., 2016). TRPV5\6 are epithelial channels with high calcium selectivity (Li, 2017). Mammalian TRPMs have eight members divided into four subgroups according to sequence homology: M1\3, M2\8, M4\5, and M6\7. Some TRPMs are distributed in the intima of cells, and their activation patterns, cation selectivity, and tissue distribution vary significantly among different TRPMs members (Kraft and Harteneck, 2005; Li, 2017; Samanta et al., 2018). TRPM is mostly cloned from cancer tissues and is related to tumor genesis, proliferation, and differentiation. They are involved in temperature sensing, Mg^2+^ homeostasis regulation, and taste conduction (Kraft and Harteneck, 2005; Samanta et al., 2018). TRPA1, the only member of the TRPA family in mammals, is an anchor-like transmembrane protein capable of sensing chemical damage (Zygmunt and Hogestatt, 2014).

**FIGURE 1:**
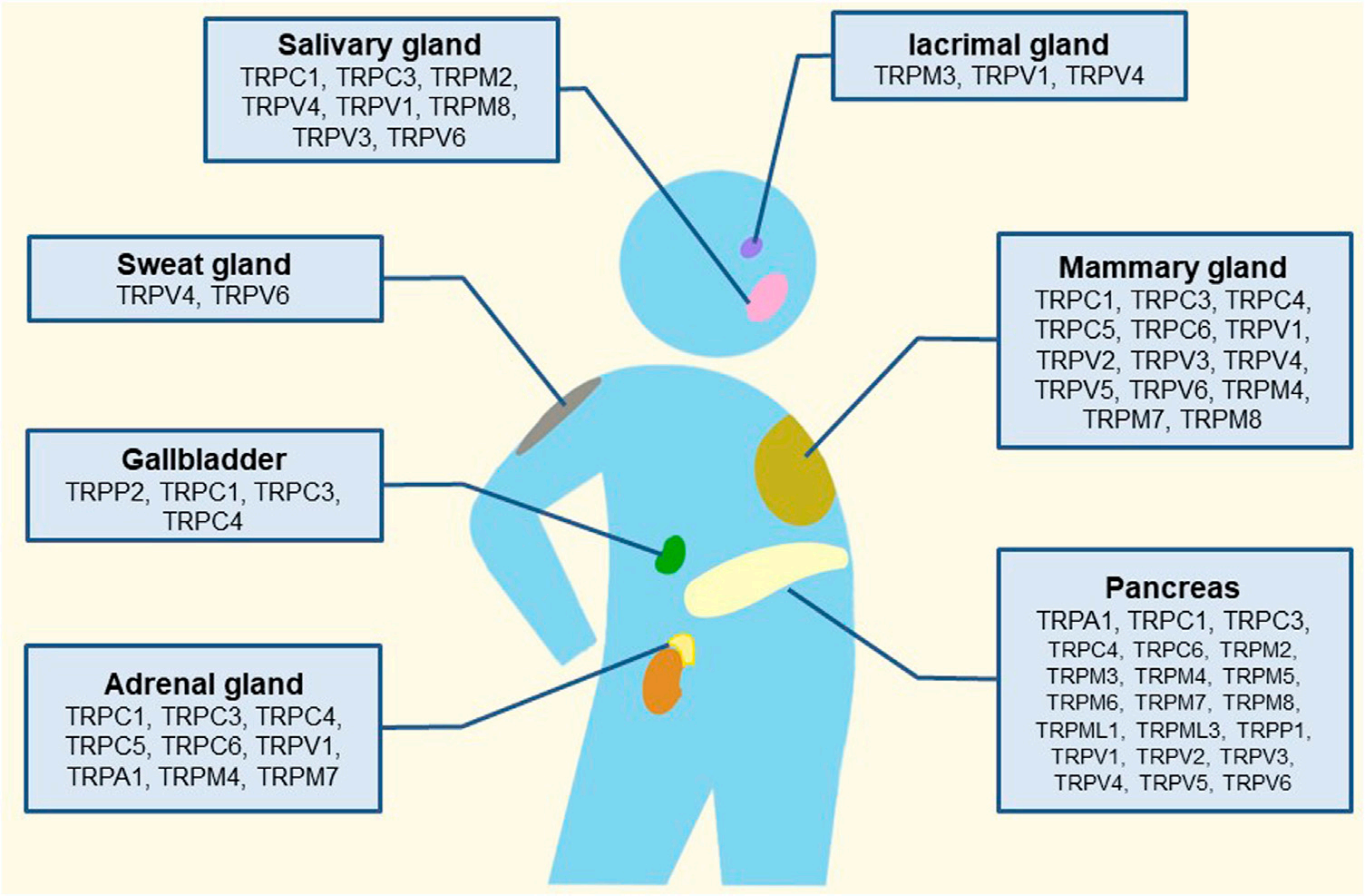
Schematic illustration of the tissue-distribution of TRP channels and their putative roles in human glands.

For TRPPs, it was found in polycystic kidney disease with the pathogenic locus of autosomal dominant and it had three members: TRPP1-3. TRPPs are composed of intact membrane proteins (Hughes et al., 1995; Mochizuki et al., 1996; Samanta et al., 2018). TRPMLs also has three members: TRPML1-3; TRP channels with the most negligible molecular weight, are associated with vesicle transport (Colletti and Kiselyov, 2011; Flores and Garcia-Anoveros, 2011; Wang et al., 2014; Venkatachalam et al., 2015). However, TRPNs do not encode in mammals, and they are related to mechanical sensory transduction in nematodes, flies, and zebrafish (Lee et al., 2010). It is worth mentioning that TRPs affect sensory function and the development of (inherited) diseases. In addition to cancers and diabetes, TRPs are often involved in heart, bone, kidney, brain, skin, eyes, and nerve diseases. TRPs are also related to the sensory transduction of chronic pain (Julius, 2013; Li, 2017; Samanta et al., 2018). Since TRPs are primarily on the surface of cells, they have also been studied as targets for drugs such as analgesics (Moran, 2018; Koivisto et al., 2022). The following review focuses on the research mechanism and drug progress of TRP channels in the pancreas, salivary gland, lacrimal gland, adrenal gland, mammary gland, gallbladder, and sweat gland (Figure 1).

## TRP Channels in the Pancreas

### TRPC Channels

As a component of the store-operated Ca^2+^ (SOC) channels, TRPC channels, primarily located in the plasma membrane, can be activated by G protein-coupled receptors (GPRs), protein kinases, or mechanical stimulation to form non-selective Ca^2+^ osmotic channels, mediating Ca^2+^ and some monovalent ions influx, and promoting cell depolarization (Chen et al., 2020; Lopez et al., 2020). In the pancreas, TRPC1, TRPC3, and TRPC4 have promoted insulin secretion by β cells. In rat β cells, TRPC1 and Orai1 are coupled to form cationic channels that mediate Ca^2+^ influx and are regulated by stromal interaction molecule 1(STIM1) (Islam, 2011; Najder et al., 2018; Islam, 2020). STIM1 gates TRPC1 through intermolecular interactions (Islam, 2011; Islam, 2020). In addition, TRPC1 is phosphorylated by protein kinase C-α (PKCα) to promote glucose-induced insulin secretion (Xu et al., 2019). Under normal circumstances, TRPC3 is activated by G protein-coupled receptor 40 (GPR40) in rat and mouse β cells. In rat β cells, Activated TRPC3 enhanced insulin secretion via phospholipase C, PKC, and GPR40, leading to membrane depolarization and Ca^2+^ influx (Hayes et al., 2013). The high expression level of TRPC3/TRPC6 induces pancreatic α and β cells proliferation via insulin-related transcription factor pancreatic and duodenal homeobox 1 (PDX-1) (Hayes et al., 2013). TRPC4 was expressed in rat and mouse islet β cells and insulinoma cells. In βTC3 cells, intracellular Ca^2+^ depletion activates TRPC4, which generates a cationic current that stimulates membrane oscillation and promotes Ca^2+^ influx, thereby increasing insulin secretion (Roe et al., 1998; Islam, 2020). Leptin, the activator of TRPC4, phosphorylates TRPC4 by phosphoinositide 3-kinase. Similarly, in INS-1 cells, protein histidine phosphatase 1 phosphorylates TRPC4, which also translocates K-ATP channels to the plasma membrane typically and promotes insulin secretion (Park et al., 2013; Srivastava et al., 2018).

In addition, in the case of PDAC, TRPC1, TRPC3, and TRPC6 may also be activated by mechanical stimuli to aggravate the disease. In the PDAC pressure microenvironment, TRPC1 transmits the mechanical signal that mediates the activation and migration of pancreatic stellate cells (PSCs) and induces chemotaxis in neutrophils. Furthermore, it was predicted that it cooperated with TRPM7 and TRPV4 to promote the progress of PDAC (Lindemann et al., 2015; Fels et al., 2016; Najder et al., 2018). In the pathological condition of PDAC, activated TRPC3 and K_Ca_3.1 (Ca^2+^-dependent K^+^ 3.1) channels in the PDAC matrix jointly promote the migration and taxis of PSCs, and the over-activation of PSCs to produce excessive extracellular matrix proteins is the leading cause of pancreatic cancer fibrosis. Moreover, in the hypoxic tumor microenvironment of PDAC, TRPC6 is not only richly expressed in PSCs but also can be activated by hypoxia, thus promoting the secretion of migration factors, which may also worsen the disease progression, which is also related to its ability to control Ca^2+^ influx (Nielsen et al., 2017; Storck et al., 2017; Najder et al., 2018).

### TRPV Channels

TRPVs are also predominantly expressed in islet β cells. Among them, TRPV2 and TRPV4 also have similar insulin-secreting effects as mentioned above (Islam, 2011; Uchida and Tominaga, 2011; Sawatani et al., 2019). However, TRPV1 transmitting neural sensation does not directly promote insulin secretion, and the role of TRPV5 and TRPV6 in islet remains to be studied. TRPV1 was expressed in INS-1E cells (Fagelskiold et al., 2012). Although TRPV1 does not directly affect glucose-induced insulin secretion in mouse β cells (Diaz-Garcia et al., 2014), it is expressed in sensory nerve fibers of the mouse pancreas, and its locus is associated with the risk of autoimmune diabetes (Razavi et al., 2006; Najder et al., 2018). During insulin release, TRPV1 is activated to promote the release of calcitonin-gene-related peptide (CGRP) and substance P (SP) by neurons, and the concentration of CGRP and SP are balanced with glucose concentration by regulating insulin concentration (Zhong et al., 2019). In nonobese diabetic mice, neuronal failure to release CGRP and SP due to TRPV1 mutations lead to physiological dysfunction of β cells, resulting in insulin resistance and β cell stress (Razavi et al., 2006; Najder et al., 2018; Zhong et al., 2019). In addition, glucagon-like peptide 1 (GLP-1) can also enhance insulin secretion, but different from the above-mentioned TRP channels, TRPV1 in the ileum is activated by capsaicin, which increases GLP-1 secretion and leads to increased pancreatic insulin secretion (Wang et al., 2012); however, this interpretation should be treated with caution. Although TRPV1 is typically excited by capsaicin, whether TRPV1 promotes insulin secretion has been highly controversial (Zhong et al., 2019). Notably, TRPV1 also expresses and mediates inflammation and pain sensation in afferents of acute pancreatitis, in coordination with TRPA1. It is possible that they regulate [Ca^2+^]_i_ and increase the release of inflammatory and pain mediators (Schwartz et al., 2011; Schwartz et al., 2013).

Ca^2+^ permeable TRPV2 is expressed in mouse insulinoma MIN6 cells and β cells (Islam, 2011). When unstimulated, TRPV2 is distributed in the cytoplasm, mainly in the endoplasmic reticulum (Uchida and Tominaga, 2011). Under high glucose conditions, insulin binds with its β cell receptor, phosphatidylinositol 3 kinase (PI3K), to stimulate the translocation of TRPV2 from the cytoplasm to membrane, which increases [Ca^2+^]_i_, accelerates insulin secretion, and stimulates β cell growth (Hisanaga et al., 2009; Aoyagi et al., 2010; Uchida and Tominaga, 2011). In addition, the osmotic swelling of cells during high glucose also activates TRPV2 to depolarize cells and increase glucose-induced insulin secretion (Sawatani et al., 2019). It was also reported that the antiaging gene Klotho translocated TRPV2 in MIN-6 (Lin and Sun, 2012).

As a permeable Ca^2+^ channel, TRPV4 is expressed in human pancreatic non-β cells and, like TRPV2, is a thermosensitive osmotic and mechanical sensor; it is also activated by the swelling of cells stimulated by glucose (Islam, 2011; Uchida and Tominaga, 2011; Marabita and Islam, 2017). Moreover, in mouse β cells, TRPV4 can be activated by sensing mechanical membrane changes induced by human islet amyloid polypeptide, which affect [Ca^2+^]_i_ and promote insulin secretion (Casas et al., 2008). In addition, the activation of TRPV4 also promotes insulin secretion in INS-1E cells, and extracellular signal-regulated kinase may be involved in this process (Skrzypski et al., 2013; Billert et al., 2017).

TRPV5/6 are the epithelial Ca^2+^ permeable channels. TRPV5 exists in secretory granules of β cells, but its effect on secretion is unknown. TRPV6 is expressed in the exocrine acinar of the pancreas, especially in human and mouse islet α cells (Hoenderop et al., 2003; Marabita and Islam, 2017). In INS-1 cells, TRPV6 regulates calcium ions to influence insulin mRNA expression and cell proliferation, but does not seem to affect insulin secretion (Skrzypski et al., 2015). Pathologically, TRPV6 overexpression in the cytoplasm of pancreatic cancer cells promotes the migration of cancer tissues (Song et al., 2018).

### TRPM Channels

TRPMs are also expressed in the pancreas. In addition to being activated by their activators, TRPM2, TRPM4, and TRPM5 can also be triggered by the influx of Ca^2+^ to participate in membrane depolarization, and TRPM2, TRPM7, and TRPM8 are involved in the progression of pancreatic adenocarcinoma (Islam, 2011; Uchida and Tominaga, 2011; Yee et al., 2012a; Uchida et al., 2017; Islam, 2020). TRPM2 is also engaged in β cell apoptosis in animals other than humans. TRPM2 was found to express in mouse and human β cells and rat INS-1 cells. TRPM2 was activated by ADP ribose (ADPR), Nicotinamide adenine dinucleotide (NAD^+^) concentration, and reactive oxygen species (ROS) to mediate Ca^2+^ influx and depolarization to regulate glucose-dependent insulin secretion (Pi et al., 2007; Du et al., 2009; Leloup et al., 2009; Islam, 2011; Islam, 2020). Particularly, TRPM2 is a calcium-permeable thermosensitive channel. Before being activated by ADPR, extracellular Ca^2+^ must be combined with the calcium sensor in the TRPM2 channel, which reflects the leading role of Ca^2+^ (McHugh et al., 2003; Csanady and Torocsik, 2009; Du et al., 2009). At a high glucose concentration, besides essential ADPR and Ca^2+^, other activators of TRPM2 include arachidonic acid produced by glucose metabolism, PKA-dependent cyclic adenosine monophosphate (cAMP), intestinal incretin hormone GLP-1, and physical second messenger “heat” (Togashi et al., 2006; Islam, 2011; Yosida et al., 2014). Growth hormone-releasing peptides Ghrelin and epinephrine inhibit TRPM2 by inhibiting cAMP, thus inhibiting insulin secretion (Yosida et al., 2014; Islam, 2020).

Moreover, TRPM2 was associated with β cell apoptosis. In the case of oxidative stress induced by free fatty acids or cytokines, TRPM2 is activated by ROS, which promotes mitochondrial destruction and leads to apoptosis. Still, human β cells resist such apoptosis (Wehage et al., 2002; Scharenberg, 2009; Li et al., 2017a; Islam, 2020). In addition, TRPM2 is distributed on the lysosomal membrane, gating Ca^2+^ and Zn^2+^ and affecting the externalization of phosphatidylserine (Mirnikjoo et al., 2009; Li et al., 2017a). In the case of pathological PADC, TRPM2 of neutrophils in tumor stroma may also be activated by ROS (Uchida and Tominaga, 2011; Faouzi and Penner, 2014).

TRPM3 has the permeability of Na^+^, Zn^2+^, and Ca^2+^, and has a strong absorption effect on Zn^2+^. In pancreatic β cells such as INS-1 cells, TRPM3, which is activated by pregnenolone sulfate (PregS), mediates Ca^2+^, Zn^2+^ influx, and induces the biosynthesis of zinc finger transcription factor Egr-1, thereby promoting glucose-induced insulin synthesis and release (Wagner et al., 2008; Colsoul et al., 2011; Mendez-Resendiz et al., 2020). Although the effect of TRPM3 is rapid and reversible, it does not participate in Ca^2+^ signaling in β cells, probably because PregS depolarizes with Na^+^ current and thus activates voltage-gated calcium channels (Islam, 2020). In addition to PregS, TRPM3 is also regulated by phosphatidylinositol 4,5-biphosphate (PIP_2_) and heat (Badheka et al., 2015).

Both TRPM4 and TRPM5 are intracellular Ca^2+^ dependent, and they are activated by increased [Ca^2+^]_i_ during insulin release (Colsoul et al., 2010; Uchida and Tominaga, 2011). Unlike TRPM2, TRPM4/5 are not Ca^2+^ permeable, but monovalent cation channels. Activated by Ca^2+^ influx, TRPM4/5 depolarize the membrane through Na + action and open voltage-gated calcium channels, thereby increasing glucose-induced insulin secretion (Islam, 2011; Uchida and Tominaga, 2011). In addition to being activated by [Ca^2+^]_i_, TRPM4/5 can also be activated by PKC, leading to membrane depolarization and increasing insulin secretion (Uchida and Tominaga, 2011; Shigeto et al., 2015; Uchida et al., 2017). TRPM4 and TRPM5 are also different. TRPM4 is expressed in both human and mouse β cells and INS-1 cells, and its depolarization mechanism is almost similar (Uchida et al., 2017). In addition to the PKC pathway, PIP2 also maintains TRPM4 sensitivity to [Ca^2+^]_i_ concentration, and TRPM4 is inhibited by glibenclamide (Colsoul et al., 2011). Particularly, in pancreatic acinar cells (PACs), the depolarization of TRPM4 inhibits Ca^2+^ influx (Diszhazi et al., 2021). TRPM4 is also involved in glucagon secretion in DTC1-6 of islet α cell line (Colsoul et al., 2011). TRPM5 was highly expressed in rat β cells and insulinoma cells but low in human β cells (Islam, 2020). Stevioside can regulate TRPM5, which may be related to its hypoglycemic effect (Steinritz et al., 2018). Interestingly, TRPM5 acts as a taste receptor and activation of sweet-taste receptors in mouse β cells is mediated by TRPM5, and TRPM5 knockout mice have less sweet taste preference and higher glucose tolerance (Larsson et al., 2015; Islam, 2020).

TRPM6 is not expressed in human β cells, while TRPM7, with the permeability of Mg^2+^, Ca^2+^, and Zn^2+^, is exceptionally abundant in human and mouse β cells, and can regulate [Mg^2+^]_i_ to promote insulin secretion (Islam, 2020). Importantly, in the case of human pathological pancreatic adenocarcinoma, TRPM7 and TRPM8 are overexpressed and are necessary biomarkers for cancer cell proliferation (Yee et al., 2011; Yee et al., 2012a). In pancreatic adenocarcinoma, sweetbread (SWD) mutations overexpress the suppressor of cytokine signaling 3a (socs3a) gene, resulting in cell cycle arrest in G0-G1 phases, inhibiting pancreatic epithelial cell growth, causing hypoplastic acini and dysmorphic ducts. In this process, TRPM7 of some cells is activated by SWD mutations, which mediates Mg^2+^ influx to inhibit socs3a, thus protecting cells from growth defects and enabling normal aging (Yee et al., 2011; Yee et al., 2012b). On the contrary, TRPM8 can prevent gene-induced senescence of cancer cells, which means that it is beneficial to the proliferation and anti-senescence of pancreatic adenocarcinoma cells and promotes the progression of the disease (Yee et al., 2010).

### TRPA1

TRPA1 is a calcium-permeable non-selective cation channel, dual thermoreceptor, which is highly expressed in rat pancreatic cells. It is activated by glycolytic products at high glucose concentrations and positively regulated by estrogens and their metabolites (Uchida et al., 2017; Mendez-Resendiz et al., 2020). In mouse islet β cells and rat INS-1 cells, TRPA1 is directly activated by hydroxylated catechol estrogens such as 2-hydroxyestradiol, which accelerates membrane depolarization and calcium influx through synergistic K-ATP channel closure, thereby increasing glucose-induced insulin secretion (Colsoul et al., 2011; Ma et al., 2019; Mendez-Resendiz et al., 2020). However, long-term stimulation of TRPA1 interferes with the transcription of insulin-related factors such as pancreatic and duodenal homeobox 1 (PDX-1) and reduces insulin secretion (Steinritz et al., 2018).

### TRPML Channels

**FIGURE 2:**
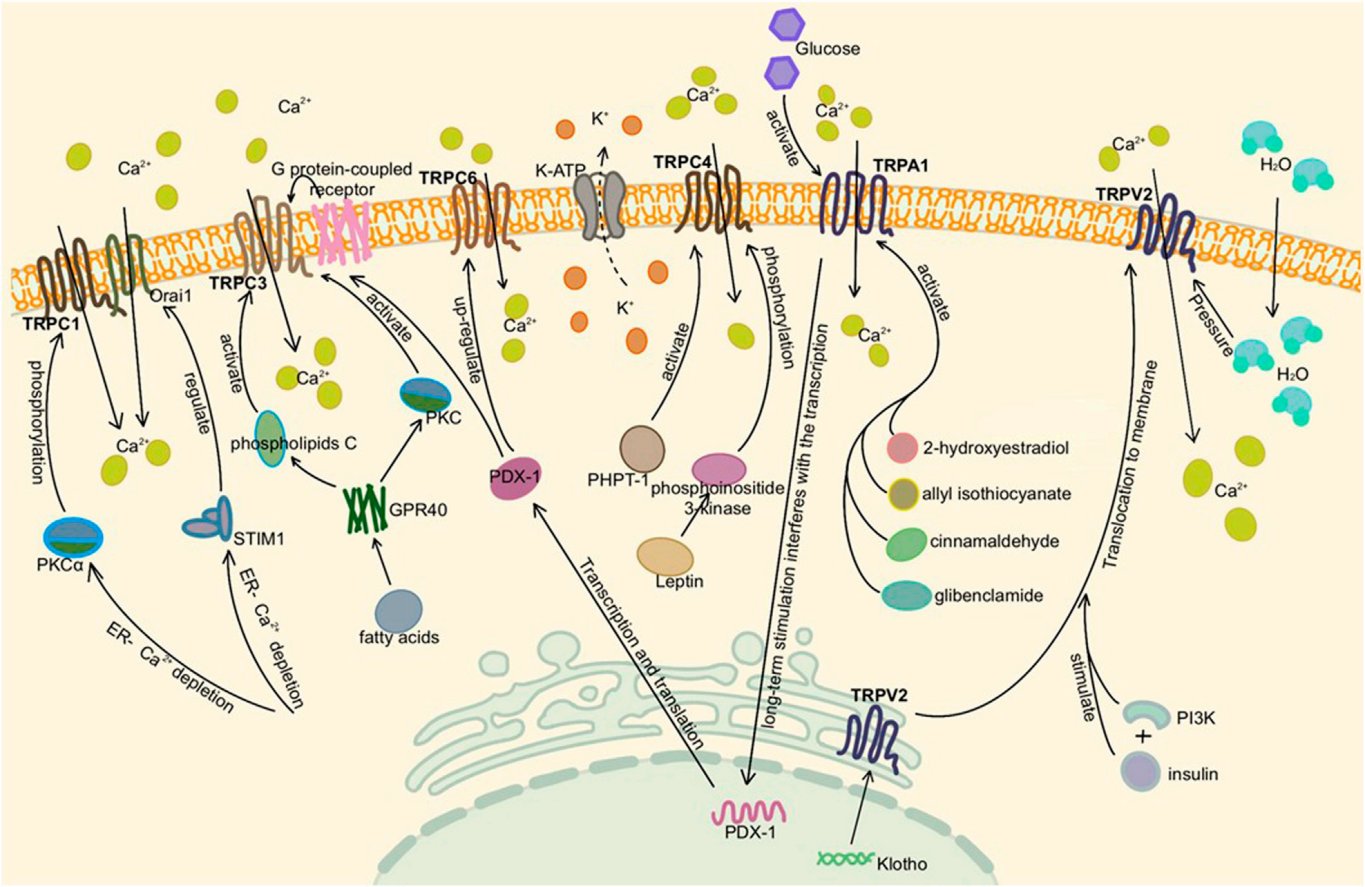
Activation of TRP channels in the pancreas. Here is pancreatic β cell or adenoma cell. TRPC1, TRPC3, TRPC4, and TRPA1 are activated by different pathways, mediating extracellular Ca^2+^ influx in the presence of Ca^2+^ deficiency in the ER and depolarizing to stimulate insulin secretion. TRPC6 is associated with cell proliferation and tumor migration. TRPV2, which existed on vesicles and ER, was activated by insulin synthesis or altered intracellular and extracellular osmotic pressure under high glucose conditions, translocated to the plasma membrane and increased Ca^2+^ influx.

**FIGURE 3:**
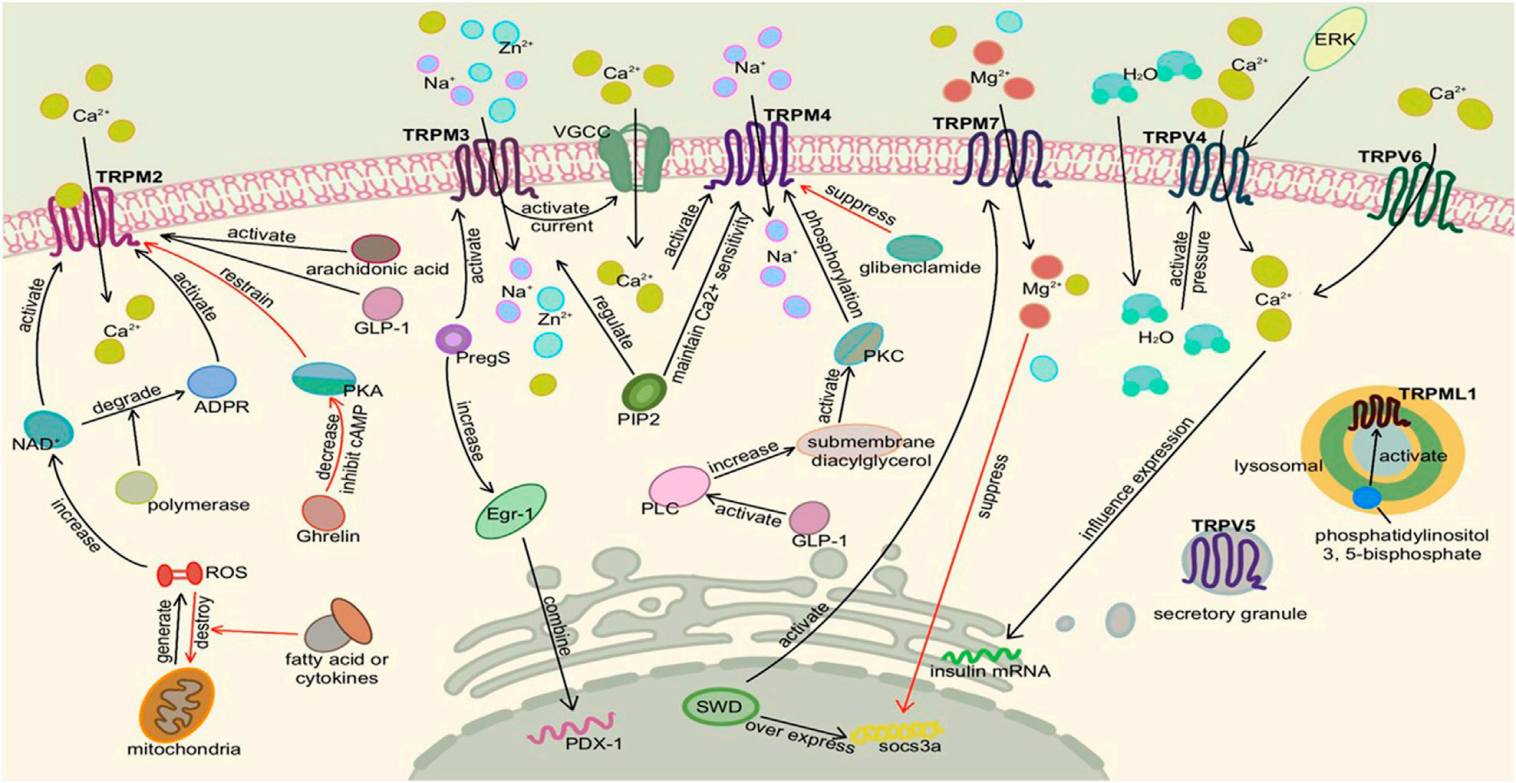
Activation of TRP channels in the pancreas Ⅱ. Here is pancreatic β cell or adenoma cell. TRPM2, TRPM3, TRPM4, and TRPM7 are also activated to promote insulin secretion. It is noteworthy that TRPM2 needs to bind to Ca^2+^, TRPM3 mainly mediates Na^+^ and Zn^2+^ influx, TRPM4 mainly mediates Na^+^ influx, and TRPM7 mediates Mg^2+^ influx. They mainly mediate extracellular Ca^2+^ influx by changing membrane potential and activating voltage-gated calcium channels. Similar to TRPV2, TRPV4 was activated by osmotic pressure under high glucose conditions. Although TRPV6 exists in INS-1 and inhibits insulin mRNA expression, it does not affect insulin secretion alone.

TRPML1, TRPML3, and TRPP1 are expressed in human pancreatic β cells and islets (Marabita and Islam, 2017). TRPML1 is also a Ca^2+^ osmotic channel, mainly located in intracellular vesicles. Since the activity of TRPML is related to pH, it tends to exist in lysosomes with low pH and can be activated by phosphatidylinositol 3,5-bisphosphate, which is rich in lysosomes. It plays a role in vesicle transport and lysosomal exocrine secretion (Li et al., 2017b; Di Paola et al., 2018). Moreover, TRPML1 mutation can lead to the loss of lysosome function (Sun et al., 2000; Dong et al., 2009). Finally, TRPP1 acts as a Ca^2+^ permeable non-selective cation channel, which may also promote β cell depolarization (Islam, 2020). The detailed molecular mechanism of TRP channels in the pancreas is shown in Figures 2, 3.

## TRP Channels in the Salivary Gland

### TRPC Channels

Salivary gland secretion depends on the increase of Ca^2+^ in salivary gland cells; when both extracellular and endoplasmic reticulum (ER) Ca^2+^ are insufficient to maintain the concentration gradient, the Store-Operated Ca^2+^ Entry (SOCE) mechanism mediates [Ca^2+^]_i_ (Ambudkar, 2016; Liu et al., 2018). Thus, ion transporters and channels such as Na^+^/K^+^/2Cl^−^ cotransporter 1 (NKCC1), Anoctamin 1 (ANO1), and K_Ca_ are opened to regulate intracellular and extracellular Cl^−^ concentration and maintain osmotic gradient, allowing water to be secreted through the water channel Aquaporin 5 (AQP5) in the apical membrane (Ambudkar, 2012; Ambudkar, 2014; Liu et al., 2018). In this process, the channels involved in SOCE are mainly TRPC1 and Orai1. Orai1 is an important component of store-operated calcium release-activated calcium and SOC channels, and the role of TRPC1 depends on the activity of the Orai1 channel (Liu et al., 2000; Liu et al., 2007; Cheng et al., 2008; Cheng et al., 2011; Hong et al., 2011; Ambudkar, 2016). Orai1 is first activated to mediate Ca^2+^ influx, which stimulates the movement of TRPC1 to the plasma membrane after the acinar cells are stimulated by neurotransmitters. Moreover the two channels are coupled into a single channel to increase Ca^2+^ influx and salivary secretion by activating an ER- Ca^2+^ binding protein- STIM1 and Caveolin-1 (Zeng et al., 2008; Pani et al., 2009; Cheng et al., 2011; Hong et al., 2011; Pani et al., 2013; Choi et al., 2014; Ambudkar, 2016; Liu et al., 2018).

Except for TRPC1, TRPC3 and TRPC4 also participate in SOCE, but whether TRPC3 participates in SOCE seems to be related to cell type and expression level (Liu et al., 2018). In salivary gland ductal cells, TRPC3 acts in a similar way to TRPC1 that TRPC3 is also associated with Orai1 after being activated by STIM1. However, TRPC3 must bind to TRPC1 before activation; that is, TRPC3 is dependent on TRPC1 to form TRPC3-TRPC1 heteromeric channel (Lee et al., 2014; Ong et al., 2014; Ambudkar, 2016). In pathological conditions, the generation of cytotoxicity in salivary acinar cells during inflammation is related to the Ca^2+^ influx mediated by TRPC3, which is reflected in ROS production and membrane damage. This phenomenon is similar with the increased of [Ca^2+^]_i_ in pancreatic acinar cells aggravating pancreatitis. It is suspected that TRPC1 is also involved in this cell damage process (Kim et al., 2011; Ambudkar, 2016).

### Other Channels

Loss of salivary gland function leads to xerostomia, often caused by radiation therapy or chronic autoimmune disease Sjögren’s syndrome (pSS), which is associated with an unregulated increase in intracellular Ca^2+^ (Konings et al., 2005; Mavragani and Moutsopoulos, 2014; Liu et al., 2018). For the loss of salivary gland function caused by pSS, pSS itself leads to the overexpression of TNF-α, interleukin, γ-IFN, and other inflammatory factors (Baturone et al., 2009; Bikker et al., 2010). Moreover, activated granulocytes also increase ROS during inflammation, which is regulated by TRPM2. TRPM2 regulates immune inflammation by regulating the release of inflammatory factors and the growth of dendritic cells (Gasser et al., 2006; Yamamoto et al., 2008; Sumoza-Toledo et al., 2011; Knowles et al., 2013).

TRPM2 is the ROS receptor existing in mouse salivary acinar cells, which may be involved in radiation-induced cell damage and pSS (Sumoza-Toledo and Penner, 2011; Liu et al., 2013; Liu et al., 2018). TRPM2 is activated by NAD+ and its ADPR generated after hydrolysis to guide Ca^2+^ influx and participate in cell damage when the radiation creates an excessive ROS microenvironment (Perraud et al., 2003; Sumoza-Toledo and Penner, 2011; Ambudkar, 2016; Liu et al., 2018). TRPM2 plays a key role in the relationship between cell damage after radiation and dry mouth (Liu et al., 2013; Teos et al., 2016; Liu et al., 2018). In conclusion, TRPM2 mediates irreversible salivary gland damage after radiation by excessive consumption of STIM1 and dysregulation of SOCE (Liu et al., 2013; Liu et al., 2017). For the recovery of damaged glands after radiation, inhibition of TRPM2 activation can promote the regeneration of glandular cells by keeping STIM1 and SOCE at normal levels (Jang et al., 2016; Liu et al., 2017). Moreover, TRPM2 was not only associated with salivary gland secretion loss, but also inhibited the abnormal shrinkage of acinar cell volume, which is related to the change of osmotic pressure during salivary gland secretion (Ambudkar, 2016). For example, TRPM2 is activated after radiation to regulate ion transporters, changes osmotic gradients, and normalizes cell size through regulatory volume increase (RVI) under the condition of being excited by agonists such as carbachol (CCh) (Liu et al., 2018). In contrast to TRPM2’s RVI, swollen cells that absorb water can return to normal size by the opposite route, known as regulatory volume decrease (RVD). TRPV4 can be activated under hypotonic conditions and play an RVD role by regulating osmotic gradient by guiding Ca^2+^ influx as well (Liu et al., 2006; Liu et al., 2018). Additionally, TRPV4 also can be activated by 4α-phorbol-12,13 didecanoate, Muscarine, GSK1016790A, and heat. Muscarine leads to salivary secretion by activating TRPV4 through heat, which opens the Cl^−^ transport channel ANO1 (Zhang et al., 2012a; Sobhan et al., 2013; Derouiche et al., 2018).

**FIGURE 4:**
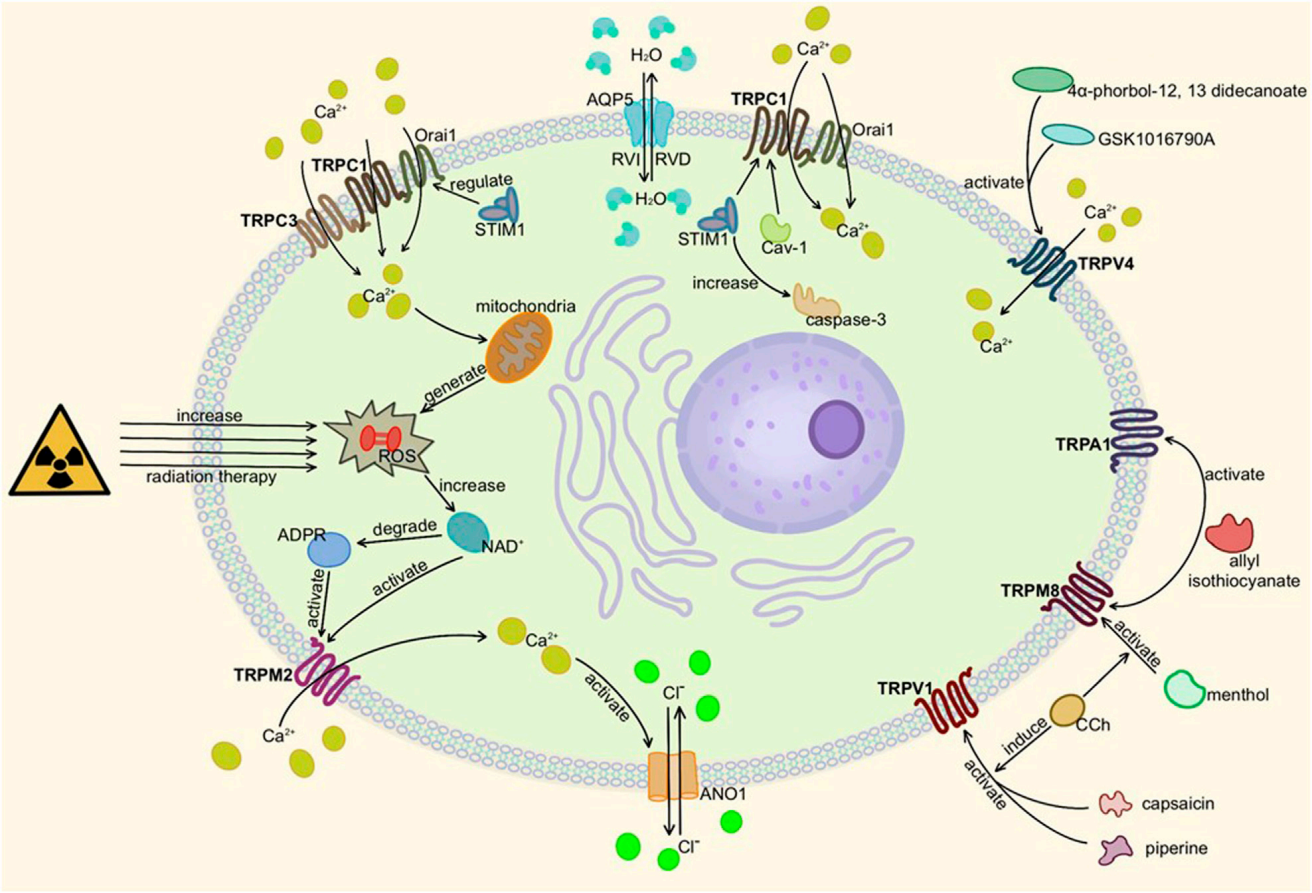
Activation of TRP channels in salivary glands. Here is salivary acinar cell (TRPC3 is present in duct cells). When TRPM2, TRPC1, TRPC3, or TRPV4 are activated and mediate Ca^2+^ influx, ANO1 and other ion transport channels can be activated to change osmotic pressure and secrete or absorb water through AQP5. TRPA1, TRPV1, and TRPM8 also affect cell secretion but remain to be studied. In addition, too much ROS can damage gland cells, resulting in secretion disorders.

Among them, TRPV1, TRPM8, TRPV3 and TRPV6 are also found in salivary glands. TRPV1 agonists-capsaicin, piperine, or TRPM8 agonist -menthol increased CCh-induced salivary secretion. Allyl isothiocyanate, the co-antagonist of TRPM8 and TRPA1, decreased the salivary secretion, indicating that the roles of these TRP channels are different (Peng, 2011; Sobhan et al., 2013; Liu et al., 2018; Houghton et al., 2020). TRPC1, TRPV4, TRPM8, and TRPV3 are also involved in the early tubular structure differentiation of salivary gland cells (Fujiseki et al., 2017). The detailed molecular mechanism of TRP channels in the salivary gland is shown in Figure 4.

## TRP Channels in the Adrenal Gland

### TRPC Channels

The adrenal gland is a vital endocrine organ of the human body. The gland is divided into two parts, the adrenal cortex and the adrenal medulla (AM). The chromaffin cells in the medulla secrete catecholamine hormones, and the cortex mainly secretes corticosteroids, including aldosterone and glucocorticoids. These hormones always affect the function of the human body, and their balance is of great significance to human health. Many subfamilies of TRP channels are expressed in the adrenal gland and play a significant role in adrenal physiology and pathology.

It has been confirmed that TRPC1, TRPC4, and TRPC5 are expressed in guinea pig AM cells at the protein level. Keita et al. found that STIM1 promotes the formation of TRPC1 with TRPC4 heteromer channels as well as insertion into the cell membrane in PC12 cells and guinea pig AM cells (Harada et al., 2019). TRPC1—TRPC4 heteromeric channels function as store-operated Ca^2+^ entry channels in endothelial cells (Sundivakkam et al., 2012). And TRPC3 (Goel et al., 2007), TRPC5 (Bezzerides et al., 2004), and TRPC6 (Cayouette et al., 2004) are inserted into the cell membrane in response to stimulation by G protein-coupled receptors (GPCRs) or receptor tyrosine kinases. TRPC1—TRPC4 heteromeric channels, therefore, mediate Ca^2+^ influx in response to stimulation of muscarinic receptors in guinea pig AM cells.

**FIGURE 5:**
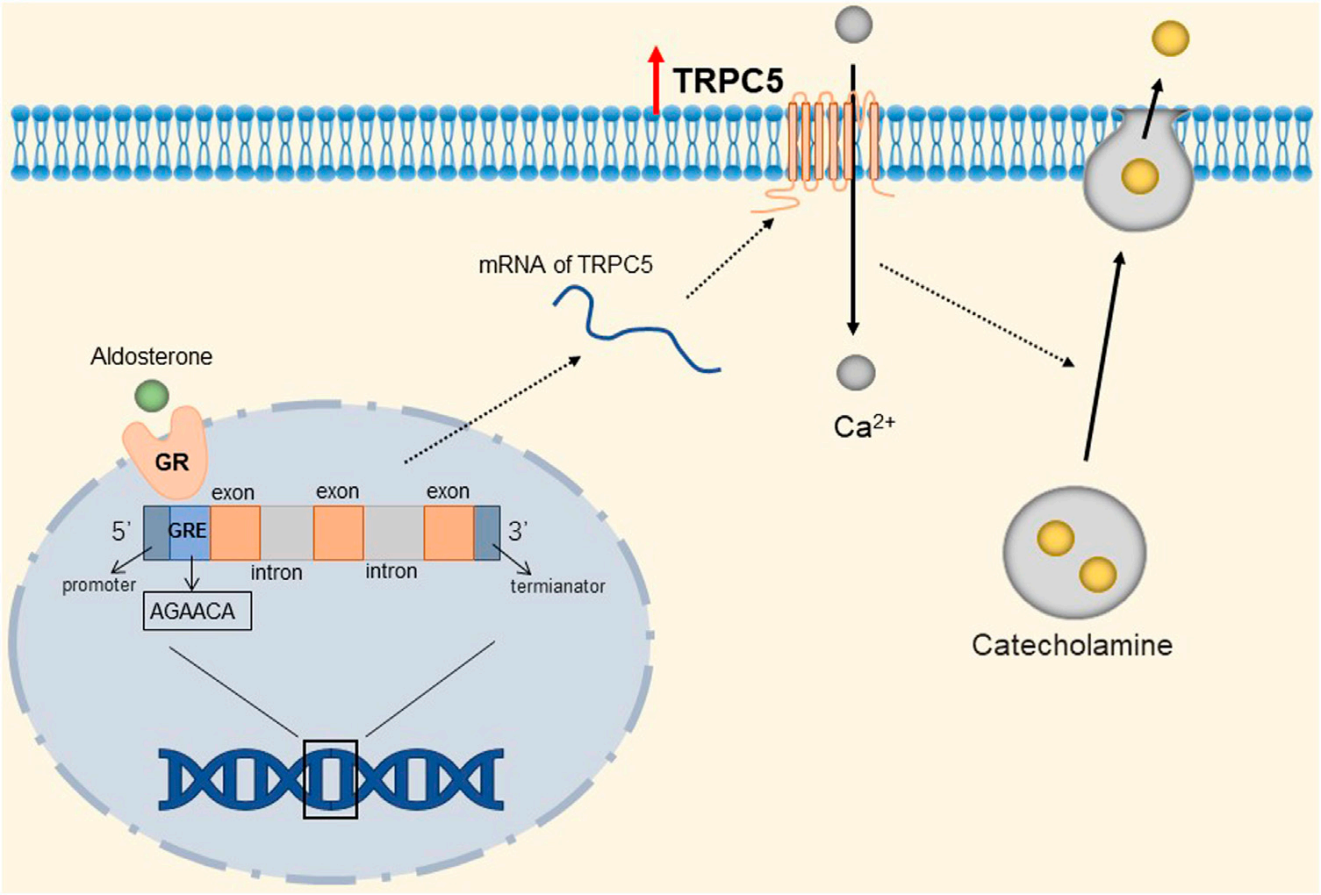
Mineralocorticoid-mediated up-regulation of TRPC5. The GRE-like half-site (AGAACA) in the TRPC5 gene binds to the mineralocorticoid receptor, prompting the up-regulation of TRPC5 channels. TRPC5 is activated to mediate the influx of Ca^2+^ into chromaffin cells, depolarizing chromaffin cells and prompting their secretion of catecholamines.

TRPC channels expression in the adrenal gland is increased in patients with metabolic syndrome, which may be a potential risk of inducing cardiovascular disease (Hu et al., 2009). Renin-angiotensin-aldosterone system activity is significantly increased in metabolic syndrome (Krug and Ehrhart-Bornstein, 19792008; Sarzani et al., 2008). High concentrations of aldosterone can regulate gene expression through corresponding receptors, which include binding to the glucocorticoid response element (GRE) half-site sequence (Olivos and Artalejo, 2008; Bhargava et al., 2004). Mineralocorticoid and glucocorticoid receptors bind to the same response element in the promoter, the GRE. Several GRE-like half-site motifs (AGAACA) were present in the putative promoter region of TRPC1, TRPC5, and TRPC6 channels. These channels are up-regulated in patients with metabolic syndrome. When these channels are activated via the G_q/11_-PLCβ pathway or Ca^2+^ store depleting pathway (Marom et al., 2011), an influx of Ca^2+^ into chromaffin cells causes depolarization of chromaffin cells and may activate voltage-gated Ca^2+^ channels to promote their secretion of epinephrine (Burgoyne et al., 1993; Cheek and Barry, 1993). The detailed molecular mechanism is shown in Figure 5. In addition, circulating hormones, such as histamine and angiotensin II, can also stimulate catecholamine exocytosis from adrenal chromaffin cells by activating voltage-independent circulating hormone-operated cation channels (Livett and Marley, 1993; Olivos and Artalejo, 2008; Sala et al., 2008). TRPC1, TRPC5, and TRPC6 are candidates for increasing hormone-induced exocrine secretion in chromaffin cells (Obukhov and Nowycky, 2002). It is well-known that excessive release of epinephrine from the adrenal gland increases the risk of myocardial infarction, cerebrovascular accident, arrhythmia, stroke, while up-regulated TRPC1, TRPC5, and TRPC6 channels are likely to cause cardiovascular disease in patients with metabolic syndrome. Therefore, these channels in the adrenal gland may be a therapeutic intervention target in patients with metabolic syndrome. However, it is not clear whether aldosterone is the only regulator of TRPC channels in the adrenal medulla, and additional molecular factors that further up-regulate TRPC channels may exist during metabolic syndrome.

### TRPV Channels

TRPV1 is a polymodal receptor activated by physical and chemical stimulation, including heat (above 43°C) and changes in pH (both acidic and alkaline), endovanilloids, such as anandamine, N-acyldopamines, and a variety of exogenous agonists, which mainly consist of capsaicin (Zsombok, 2013; Moran and Szallasi, 2018). TRPV1 was upregulated in chromaffin cells of neuropathic animals (Arribas-Blázquez et al., 2019). Meanwhile, Pablo et al. found that stress significantly increased the mRNA levels of TRPV1 channels in adrenal cells (Surkin et al., 2018). TRPV1 is a ligand-gated, non-selective cation channel with high permeability to Ca^2+^ and therefore Ca^2+^ influx in adrenocortical cells when TRPV1 is activated (Moran and Szallasi, 2018). High levels of intracellular Ca^2+^ concentration may suppress glucocorticoid production (Matthews and Saffran, 1973). Capsaicin inhibits lipopolysaccharide-induced intracellular steroidogenesis in adrenocortical cells by activating TRPV1 and increasing intracellular Ca^2+^ levels, which may prevent the development of central nervous system disorders in patients with severe sepsis (Ferreira et al., 2019). In summary, TRPV1 channels in the adrenal gland are upregulated when the body is under stress or neuropathological conditions. Excessive expression of TRPV1 after activation by its agonists may attenuate the release of corticosteroids to some extent and thus protect the body. Finally, TRPV1 is a driver to increase the rapid release of corticosterone and epinephrine. Dekel et al. proposed an alternative strategy to modulate peripheral organ function (Rosenfeld et al., 2020). They developed a magnetothermal switch for the on-demand release of the adrenal hormones epinephrine and corticosterone by controlling TRPV1 activation *in vitro*. They use this approach to control adrenal hormone secretion in genetically intact rats wirelessly. Since alterations in the levels of these hormones are associated with psychiatric disorders such as post-traumatic stress disorder and depression, their approach may be helpful to study the physical and psychological effects of stress. While TRPV1 (and other thermosensitive TRP channels) was shown to be expressed in other organs deep in the body, such as peripheral nerves, gastrointestinal tract, pancreas, and heart (Akbar et al., 2008; Schwartz et al., 2011; Randhawa and Jaggi, 2017), the potential application of magnetothermal deep organ stimulation as means to study organ function paves the way for the development of bioelectronic medicines.

### TRPA1 Channel

TRPA1 is a Ca^2+^ -permeable non-selective cation channel that can be activated by various toxic or irritating substances in nature. Allyl isothiocyanate (AITC) and cinnamaldehyde (CAN) increase epinephrine secretion by activating TRPA1-expressing adrenal sympathetic nerves in rats (Iwasaki et al., 2008). TRPA1 is frequently coexpressed with TRPV1, raising the possibility that TRPA1 and TRPV1 mediate the function of a class of polymodal nociceptors (Liedtke and Heller, 2007). Yuriko et al. found that oleuropein aglycone (OA) stimulates norepinephrine secretion by activating TRPA1 and TRPV1, thereby enhancing UCP1 receptor expression in brown adipose tissue (BAT) (Oi-Kano et al., 2017). BAT is the organ responsible for heat production in the human body, and activation of adrenergic receptors expressed in BAT can increase UCP1 receptor expression (Himms-Hagen et al., 1994; Nagase et al., 1996; Cannon and Nedergaard, 2004). The activation of TRPV1 is a key link in capsaicin-induced epinephrine secretion. Capsaicin improves body energy metabolism by promoting catecholamine secretion and up-regulating UCP1 receptor in rats (Kawada et al., 1986; Watanabe et al., 1987; Watanabe et al., 1988; Kobayashi et al., 1998). Also, AITC increases body heat production and expression of UCP1 receptors by activating TRPA1 channels (Yoshida et al., 1988). We hypothesize that TRPA1, when expressed alone, can be activated by its agonist AITC, thereby inducing catecholamine secretion from the adrenal gland. Activation of TRPA1 is therefore likely to be one of the pathways by which the body increases energy metabolism as well as increases heat production, but there is currently insufficient evidence to elucidate the mechanism by which TRPA1 mediates catecholamine secretion after activation. However, not all TRPA1 activation can mediate the increased activity of adrenal sympathetic nerves. Some studies have found that β-eudesmol inhibits adrenal sympathetic nerve activity (ASNA) by activating TRPA1 (Ohara et al., 2018). Kazuaki et al. identified three key amino acid residues in TRPA1, namely threonine 813, tyrosine 840, and serine 873, which can be activated by β-eudesmol (Ohara et al., 2015).

### TRPM Channels

**FIGURE 6:**
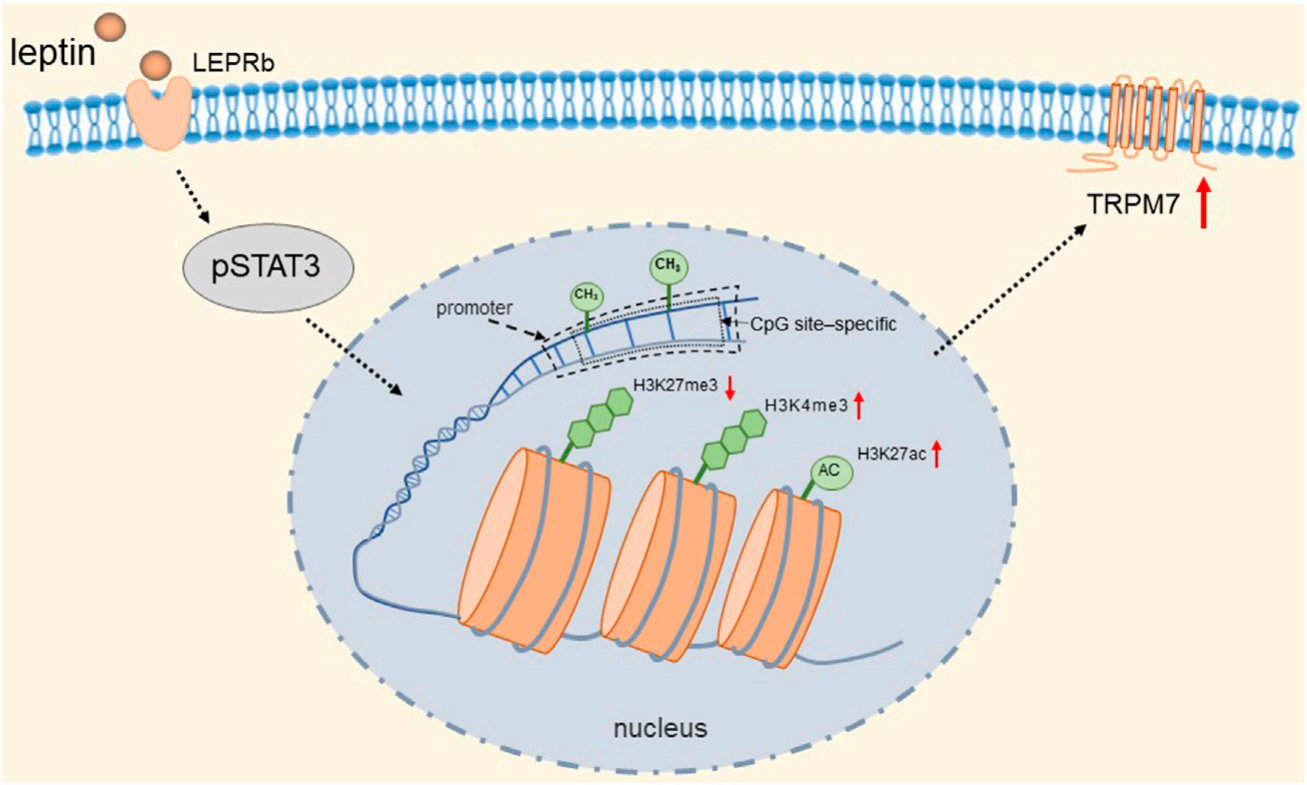
Leptin induces epigenetic regulation of TRPM7. Activated pSTAT3 epigenetically regulates transcription of TRPM7 through DNA methylation and histone modifications. Specifically, it is manifested in a reduction in CpG site-specific methylation and H3K27 trimethylation and an increase in H3K27 acetylation and H3K4 trimethylation at the TRPM7 promoter.

TRPM7 has a very broad tissue distribution as a member of the TRP channel superfamily. Its gene encodes a persistently open calcium channel regulated by intracellular Mg^2+^/ATP. It has been shown that calcium influx caused by TRPM7 plays a key role in the survival of cells (Nadler et al., 2001). Bonnie et al. found that rat adrenal pheochromocytoma (PC12) cells expressing the receptor (LEPRb) showed significant induction of promoter activity and TRPM7 expression after leptin treatment (Yeung et al., 2021). They found that activated pSTAT3 epigenetically regulates the transcription of TRPM7 through DNA methylation and histone modifications. Specifically, it is manifested in a reduction in CpG site-specific methylation and H3K27 (H3 [histone 3] K27 [lysine 27]) trimethylation and an increase in H3K27 acetylation and H3K4 (H3 lysine 4) trimethylation at the TRPM7 promoter. The detailed molecular mechanism is shown in Figure 6. Therefore, TRPM7 channels may be up-regulated when plasma levels are increased *in vivo* due to obesity, and we speculated that when this event occurs in adrenal chromaffin cells, more Ca^2+^ influx due to up-regulation of TRPM7 channels may induce increased catecholamine secretion, which is likely to be one of the reasons associated with hypertension. Fortunately, epigenetic changes are reversible, and epigenetic modifications targeting TRPM7 may serve as a novel therapeutic approach for the treatment of obese hypertension.

TRPM4 has been demonstrated to be expressed in mouse adrenal medulla (AM) cells at the mRNA levels (Mathar et al., 2010a). Masumi et al. found that pituitary adenylate cyclase-activating polypeptide (PACAP) induced catecholamine secretion in mouse AM cells, which may be due to the generation of depolarizing inward currents caused by TRPM4 channel activation (Inoue et al., 2020). Application of the endogenous PKC activator 1-oleoyl-2-actyl-sn-glycerol mimics PACAP activation of Na^+^ -permeable cation channels in bovine AM cells (Tanaka et al., 1996), and therefore, PACAP may also activate TRPM4 in an AM-cell PKA-dependent manner. Differently, PACAP does not directly induce the secretion of catecholamines in guinea-pig AM cells, but rather promotes muscarinic receptor-mediated activation of Ca^2+^-activated nonselective cation (NSC) channels, and this promotion is mainly due to the increased insertion of heteromeric TRPC1-TRPC4 channels into the cell membrane (Harada et al., 2019). Ilka et al. found that mice with Trpm4 gene deletion lost long-term regulation of blood pressure stability, which they concluded was caused by elevated plasma levels of epinephrine and norepinephrine after excluding the cause of altered cardiac function (Mathar et al., 2010b). However, the molecular mechanism of how TRPM4 regulates or promotes release from chromaffin cells remains to be clarified but may include direct dependence on TRPM4 to regulate vesicle release, as shown for another TRPM7 in cholinergic synaptic vesicles (Krapivinsky et al., 2006). These results suggest that TRPM4 activity may be beneficial in limiting elevated blood pressure in hypertensive patients. Hypertension is a potential risk factor for cardiovascular disease. In most cases, its pathogenesis remains unclear. The above lists two TRPM channels that affect the body’s blood pressure level by changing the secretion of catecholamines by adrenal chromaffin cells, demonstrating the feasibility of TRP channels as a therapeutic target for hypertension, but there are still not many studies in this area and the mechanism by which TRP channels regulate blood pressure needs to be further explored.

## TRP Channels in the Mammary Gland

The mammary gland is an accessory gland to the skin. Adult women have no secretory activity of the mammary gland when they are not pregnant, called the quiescent mammary gland. Mammary gland hyperplasia during pregnancy and exuberant secretion during lactation are called active mammary glands. TRP channels are expressed in normal mammary epithelial cell, and play an important role in Ca^2+^ transport in mammary epithelial cells. Anantamongkol et al. found that the expression of TRPC1, TRPC5, and TRPC7 increased during early lactation (Anantamongkol et al., 2009). Mammalian TRPC channels can be activated by receptor-operated or store-operated mechanisms to mediate Ca^2+^ influx, thereby prompting breast milk secretion (Ambudkar, 2006). It is therefore likely that TRPC channels that are upregulated prepare for extensive and sustained epithelial cell activity to support milk production and/or secretion. However, another study reported decreased expression of TRPC1 during lactation and early mammary involution stages in mice (McAndrew et al., 2011). VanHouten et al. showed that TRPV5 expression was lower in mammary epithelial cells during pregnancy and lactation than in quiescent mammary epithelial cells. In addition, TRPV6 is expressed in the mammary gland of quiescent mice, but its expression shows a decreasing trend throughout pregnancy and is not expressed during lactation (VanHouten and Wysolmerski, 2007).

### TRPV Channels

**FIGURE 7:**
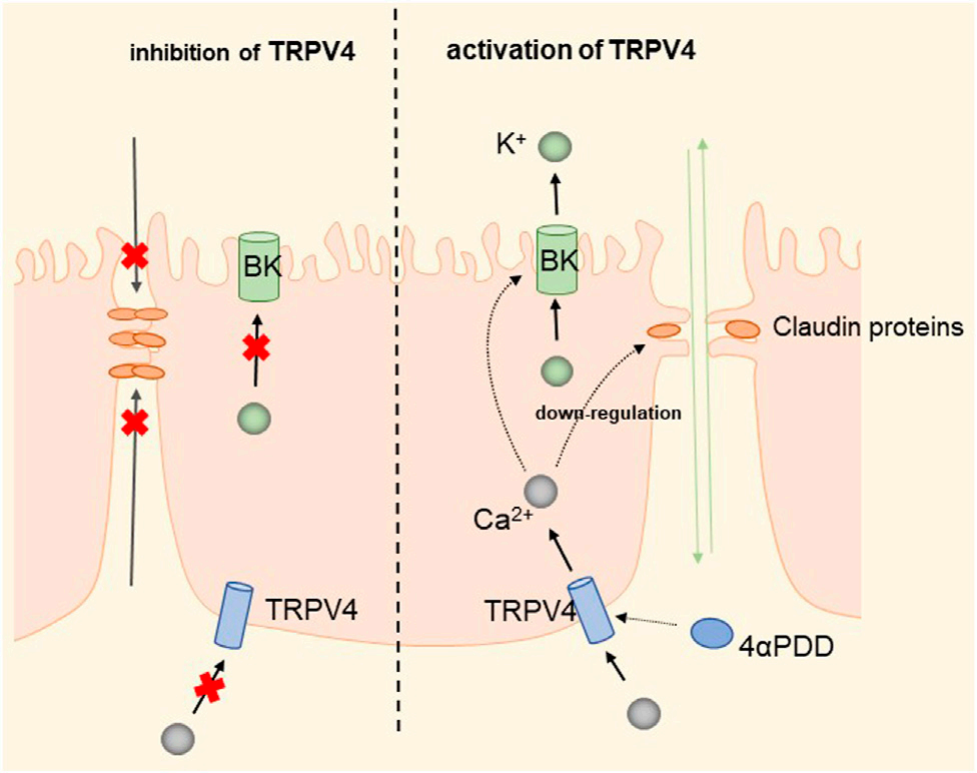
Regulation of transcellular and paracellular pathways by TRPV4 in HC11 cells. TRPV4 induces Ca^2+^ influx by activation by 4-PDD, which activates BK channels to provide transcellular ion channels. The process down-regulates sealed claudin proteins and alters the structure of tight junctions, increasing paracellular permeability and facilitating milk production.

**FIGURE 8:**
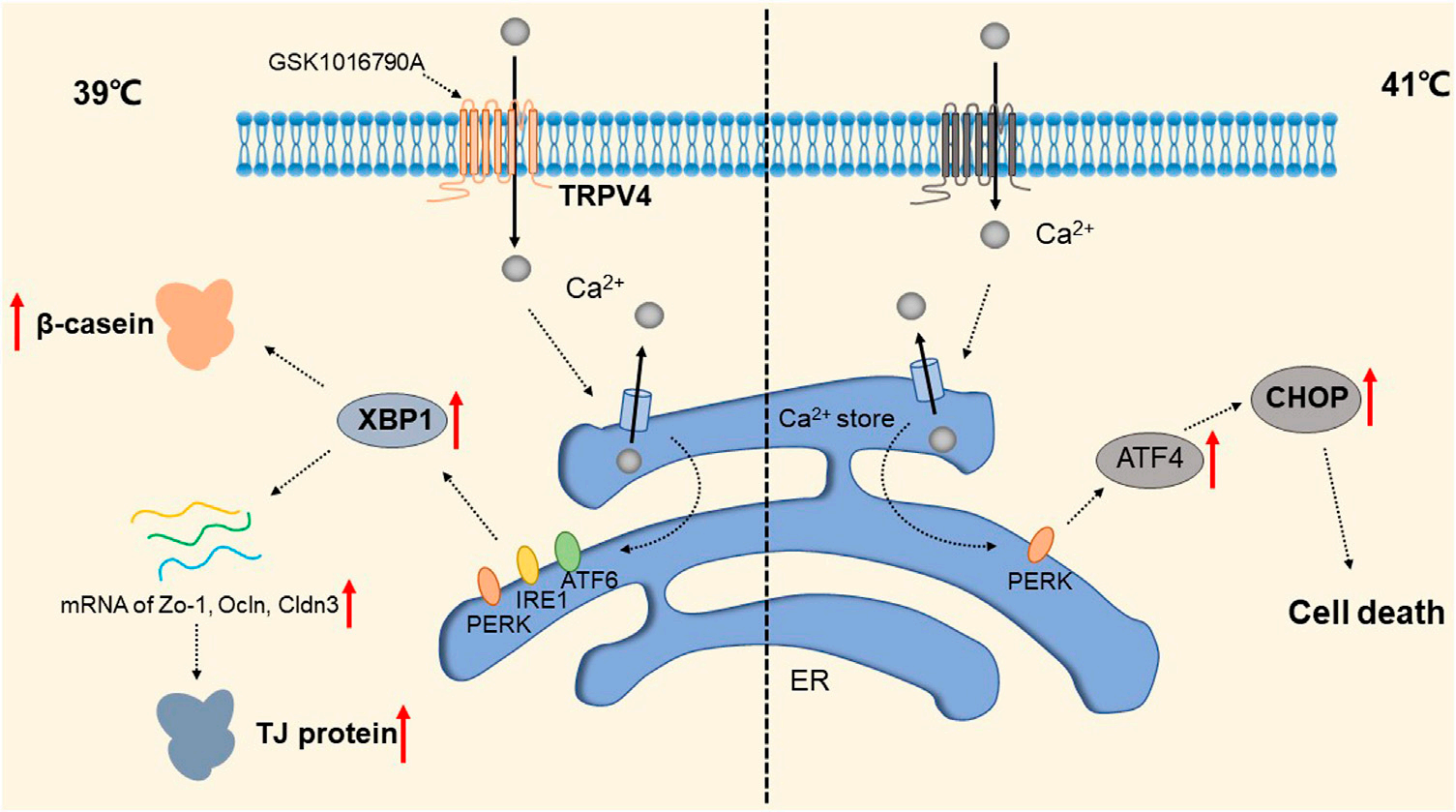
Regulation of TRP Channels on breast cells at different temperatures. The activity of TRPV4 increased at 39 °C. Activation of TRPPV4 by GSK1016790A leads to Ca^2+^ influx, promotes Ca^2+^ release from the ER, and depletes Ca^2+^ stores, at which point the UPR is initiated to upregulate transcript levels of Xbp1s via the PERK, IRE1, and ATF6 signaling pathways. This increased the expression of Zo-1, occln and Cldn3 mRNA and enhanced the expression of β-casein and TJ protein-coding genes. The presence of a certain TRP channel up-regulates the apoptosis transcription factor CHOP and promotes apoptosis through the UPR pathway at 41 °C.

The widely distributed TRPV4 cationic channel participates in the transduction of mechanical and/or osmotic stimuli in different tissues (Liedtke and Friedman, 2003; Gao et al., 2003). TRPV4 is selectively localized in the basolateral membrane compartment, where it is activated by 4-a-phorbol12,13-didecanoat (4a-PDD) (Vriens et al., 2007), resulting in calcium entry, which on the one hand activates Ca^2+^-activated K^+^ channels (BK) and provides a transcellular ion pathway. On the other hand, it increases the permeability of mammary epithelial cells, which is created by a down-regulation of sealing-type claudin proteins accompanied by an altered tight junction structure (Reiter et al., 2006). The detailed molecular mechanism is shown in Figure 7. Md Aminul et al. found that both low permeability tight junctions of mammary epithelial cells and milk production were enhanced after a mild heat treatment at 39°C. TRPV4 activity was increased during mild heat treatment at 39°C. Activation of TRPV4 by GSK1016790A leads to Ca^2+^ influx, promotes Ca^2+^ release from the endoplasmic reticulum (ER), and depletes Ca^2+^ stores, at which point the unfolded protein response (UPR) is initiated and upregulates the transcript levels of Xbp1s through PERK, IRE1, and ATF6 receptor-mediated signaling pathways (Harding et al., 2000; Celli et al., 2011; Shen et al., 2019). Upregulation of the Xbp1 gene increases the expression of β-casein (β-casein is a differentiation marker of HC11 cells and is a representative milk protein), Zo-1, ocln, and Cldn3 mRNA, and thereby regulates the expression of TJ protein-coding genes. Interestingly, TRPV4 expression is increased during pregnancy but decreased during lactation (Islam et al., 2020). Mammary epithelial cells are exposed to temperatures above body temperature during lactation due to metabolism (Berman et al., 1985). Heat stress induces ER stress and causes UPR (Xu et al., 2011; Kim et al., 2013). Elevated transcript levels of Chop decrease the viability of cells and therefore cause reduced lactation (Urra et al., 2013). The detailed molecular mechanism is shown in Figure 8. TRP channels cover the temperature sensing range, TRPV4 is activated at 39°C, but there may be other TRPs that are activated at higher temperatures, and although there is no evidence so far, it provides direction and ideas for later studies.

TRPV6 is expressed in normal breast and breast cancer cell lines. A positive correlation between expression of the transcription factor zinc finger homeobox 3 (ZFHX3) and promoter activity of TRPV6 was found by Dan et al. (Zhao et al., 2019). Previous studies have shown that Ca^2+^ influx mediated by TRPV6 activation is able to induce keratinocyte differentiation (Lehen’kyi et al., 2011; Lehen’kyi et al., 2007). Ca^2+^ has been shown to be involved in controlling the growth of breast cancer cells through its interaction with calmodulin (Strobl et al., 1995). TRPV6 channel-mediated Ca^2+^ influx is involved in cell proliferation, which may be associated with breast cancer cell migration (Bolanz et al., 2008; Bolanz et al., 2009).

### TRPC Channels

**FIGURE 9:**
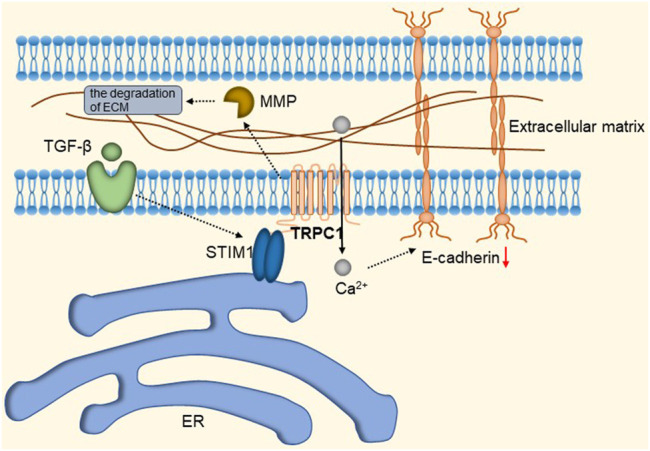
TRPC1-STIM1 complex mediates TGF-β-induced EMT. STIM1 aggregates to the endoplasmic reticulum-plasma membrane (ER-PM) junctions, forming the TRPC1-STIM1 complex with TRPC1, mediating the down-regulation of E-cadherin and leading to the activation of calproteinases and MMPs, which target proteins involved in cell adhesion and promote their migration.

**FIGURE 10:**
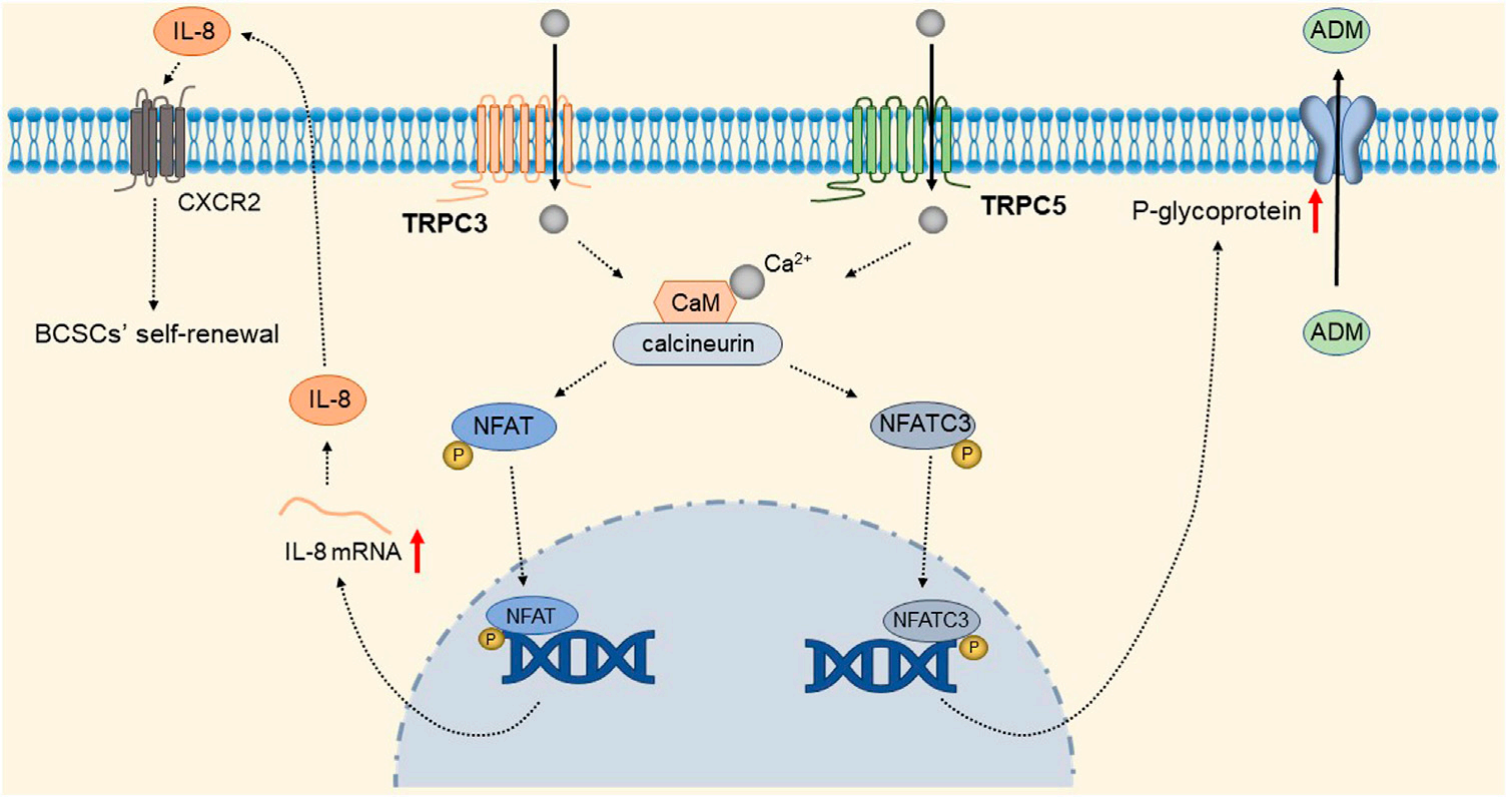
TRPC3 and TRPC5 mediate breast cancer cell proliferation and drug resistance. Ca^2+^ entry through TRPC3 and TRPC5 channels, via calmodulin-calcineurin dependent NFAT signal pathway, upregulating IL-8 and P-gp expression respectively. IL-8 mediates BCSC proliferation through CXC receptor 2. Up-regulated P-gp expels intracellular ADM into the extracellular matrix, leading to drug resistance.

TRPC1 expression is increased in invasive ductal carcinoma (Guilbert et al., 2008; Dhennin-Duthille et al., 2011; Mandavilli et al., 2012). TGFβ-induced Epithelial-to-Mesenchymal Transition (EMT) is dependent on Ca^2+^ entry via the TRPC1- Stromal Interaction Molecule 1 (STIM1) complex that leads to the activation of calpains and matrix metalloproteinases (MMPs), which target proteins involved in cellular adherence and promote their migration (Schaar et al., 2016). The detailed molecular mechanism is shown in Figure 9. Therefore, inhibition of the TRPC1-STIM1 complex may be an attractive target for the treatment of breast cancer metastasis. Naoya et al. found that LPA/LPAR3 signaling and subsequent TRPC3 activation leaded to an increase in the number of breast cancer stem cells (BCSCs) through calcium-dependent transcriptional activation of IL-8 by NFAT (Hirata et al., 2022). Polyunsaturated fatty acids (PUFA) and TRPC3 antagonists continuously inhibit the proliferation and migration of breast cancer cells (Zhang et al., 2012b), but the mechanism is still unclear. As one of the biomarkers of breast cancer development and migration, TRPC3 represents a potential target for a new class of anticancer drugs. In the future, specific therapeutic approaches to reduce tumorigenesis may be established by targeting the LPA/LPAR3 pathway and TRPC3 channels. Overexpression of TRPC5 induces chemoresistance by up-regulating of P-glycoprotein (P-gp) and hypoxia-inducible factor-1α in chemoresistant breast cancer cells (Zhu et al., 2015; Ma et al., 2012a). TRPC5-mediated Ca^2+^ entry stimulates P-gp overproduction through NFATc3 in breast cancer cells (Ma et al., 2012b). P-gp expels intracellular Adriamycin (ADM) into the extracellular matrix, leading to drug resistance. The detailed molecular mechanism of TRPC3 and TRPC5 is shown in Figure 10. In addition, TRPC5 mediates cytoprotective autophagy through the CaMKKβ/AMPKα/mTOR pathway, causing drug resistance in breast cancer cells (Zhang et al., 2017). Inhibition of P-gp activity and avoidance of autophagy is therefore an attractive approach to overcome multidrug resistance in cancer chemotherapy, and TRPC5 is a valuable target.

## TRP Channels in the Gallbladder

The gallbladder is an exocrine digestive gland of the human body that mainly serves to concentrate and store bile. The contractile emptying of the gallbladder is regulated by hormones, and cholecystokinin stimulates contraction of the gallbladder smooth muscle (GBSM) layer and excretion of bile after eating. TRPP2 channel protein belongs to the superfamily of transient receptor potential (TRP) channels and is widely expressed in the smooth muscle of the digestive tract, including gallbladder smooth muscle. TRPP2 can not only mediate intracellular Ca^2+^ release from Ca^2+^ stores, but also regulate extracellular Ca^2+^ influx and enhance intracellular Ca^2+^ concentration ([Ca^2+^]_i_) (González-Perrett et al., 2001; Koulen et al., 2002).

**FIGURE 11:**
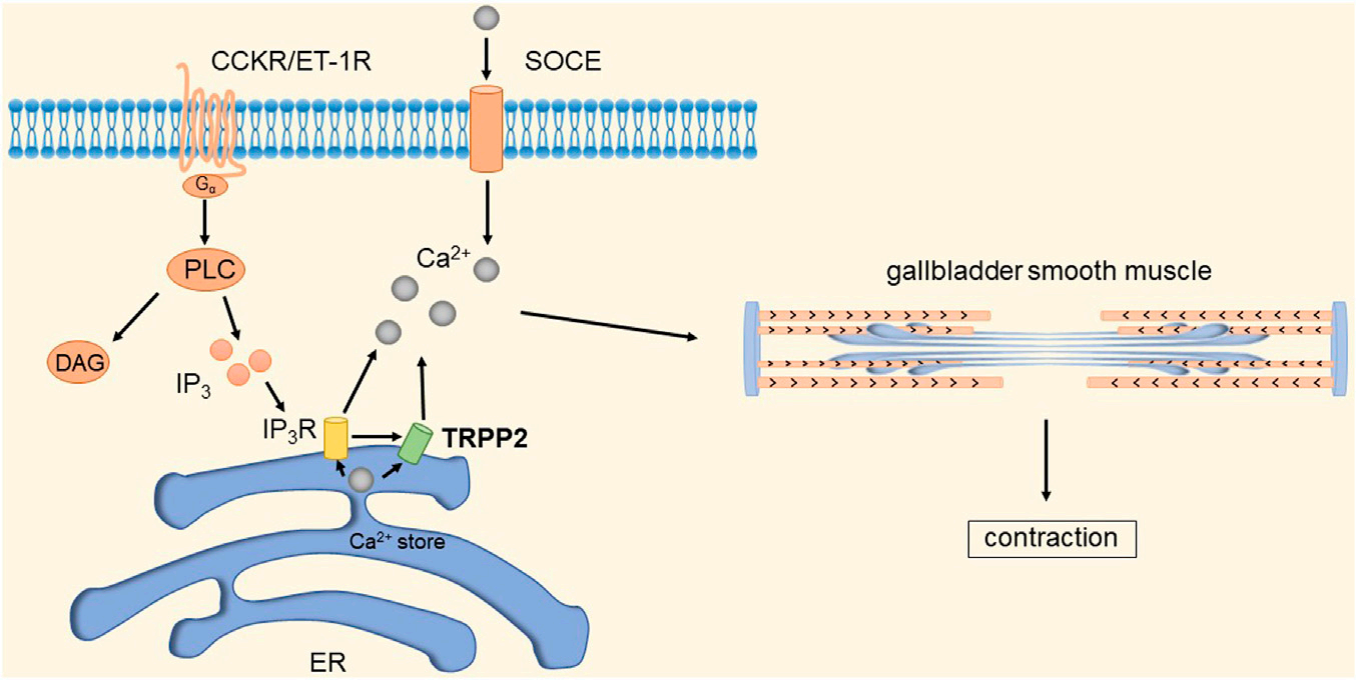
TRPP2 mediates gallbladder smooth muscle contraction. CCK and ET-1 receptors can activate PLC and guide the hydrolysis of phosphatidylinositol 4,5-bisphosphate to produce diacylglycerol and IP3. IP3 increases local [Ca^2+^]_i_ by activating IP3R to release Ca^2+^ from Ca^2+^ stores, which in turn activates TRPP2 to cause further release of Ca^2+^. Depletion of Ca^2+^ stores will subsequently initiate SOCE. The increase in [Ca^2+^]_i_ causes contraction of gallbladder smooth muscle.

It has been shown that TRPP2 channels are widely distributed in the plasma membrane and endoplasmic reticulum (ER) (Tsiokas, 2009). Xingguo et al. found that knockdown of TRPP2 protein in GBSM of guinea pigs significantly decreased Ca^2+^ release and extracellular Ca^2+^ influx evoked by carnosine (CCh), thereby inhibiting endothelin-1 (ET-1) and cholecystokinin-induced gallbladder contraction (Zhong et al., 2016). Cholecystokinin (CCK) and ET-1 receptors play an important role in GBSM contraction. CCK and ET-1 receptors, as members of the G protein-coupled receptor (GPCR) superfamily, can activate phospholipase C (PLC) and guide the hydrolysis of phosphatidylinositol 4,5-bisphosphate to produce diacylglycerol and inositol 1,4,5-triphosphate (IP3) (Noble et al., 1999). IP3 increases local cytosolic Ca^2+^ concentration by activating the IP3 receptor to release Ca^2+^ from Ca^2+^ stores, which may activate TRPP2 causing the further release of Ca^2+^ (Li et al., 2005; Sammels et al., 2010). Depletion of Ca^2+^ stores will subsequently initiate SOCE. An increase in [Ca^2+^]_i_ is a key event in eliciting smooth muscle contraction in response to agonists (Karaki et al., 1997). In addition, studies have shown that TRPP2 is also present in the plasma membrane and mediates Ca^2+^ influx (Du et al., 2012; Narayanan et al., 2013). In summary, TRPP2-mediated Ca^2+^ release expressed on the ER membrane plays an important role in agonist-induced GBSM contraction. GPCRs are key points in initiating TRPP2 opening through the PLC-IP3 pathway. The detailed molecular mechanism is shown in Figure 11.

However, in addition to TRPP2 channels, many other ion channels similarly mediate smooth muscle Ca^2+^ homeostasis and contractility. An example is a physical and functional interaction between TRPC1 or TRPC4 channels and STIM1 thought to contribute to SOCE (Sours-Brothers et al., 2009). TRPC4 and TRPC6 channels depolarize muscarinic receptor-coupled intestinal smooth muscle cells and voltage-activated Ca^2+^ influx and contraction, thereby accelerating small intestinal motility *in vivo* (Tsvilovskyy et al., 2009). Sara Morales et al. found that guinea pig gallbladder smooth muscle contains mRNAs encoding TRPC1, TRPC2, TRPC3, and TRPC4 proteins whose abundance depends on cytosolic Ca^2+^ concentration. They found that lowering the level of cellular Ca^2+^ with the use of Ca^2+^ chelators such as EGTA and BAPTA AM leads to a decrease in the expression of all TRPC members found in GBSM, whereas elevations in cellular Ca^2+^ as a result of Ca^2+^ influx or store depletion leads to an increase in TRPC gene expression (Morales et al., 2007). Therefore, we cannot deny that there are other ion channels mediating Ca^2+^ homeostasis in gallbladder smooth muscle cells, and the interaction between these ion channels still needs to be explored in the future, and the detailed mechanism of gallbladder smooth muscle contraction still needs to be further elucidated.

## TRP Channels in the Sweat Glands

Human skin has more than two million sweat glands and plays a great role in excreting waste products and maintaining water and salt balance. Sufficient studies have shown that TRP channels are abundantly expressed in the skin, and various TRP channels are involved in the formation and maintenance of the skin barrier, the growth of HF, and the immune and inflammatory processes of the skin, thereby maintaining skin homeostasis and promoting the occurrence of a variety of skin diseases. More importantly, several skin-expressed TRP channels act as primary sensors for temperature, mechanical, and chemical stimuli, modulating our temperature, touch, itch, and pain perception under physiological and pathological conditions. TRPV4 channels are present in cutaneous vascular endothelial cells and eccrine secretory cells (Kida et al., 2012; Fusi et al., 2014; Olivan-Viguera et al., 2018), and activation of the channel increases the concentration of intracellular Ca^2+^. If it has an important function in the cell types described above, it leads to vasodilatation (Félétou and Vanhoutte, 2009) and sweat secretion (Sato and Sato, 1981; Metzler-Wilson et al., 2014). Naoto et al. showed that TRPV4 channel activation mediates cutaneous vasodilatation but has no effect on sweating and that activation of TRPV4 channels alone is not sufficient to induce sweat secretion in humans. However, TRPV4 channel activation may increase calcitonin gene-related peptide (Gao and Wang, 2010), a peptide that has been shown to increase muscarinic sweating in humans (Schlereth et al., 2006). TRPV6 is involved in the uptake of ca ions and regulates intracellular ca ion levels in keratinocytes. Extracellular ca ion-induced differentiation up-regulated the mRNA and protein levels of TRPV6, and keratinocyte differentiation was affected after siRNA silencing of TRPV6 in human primary keratinocytes (Lehen’kyi et al., 2007). However, there are no excessive studies to elucidate the link between TRP channels and sweat glands, and there may be other TRP channels other than TRPV4 that mediate the secretion of sweat glands.

## TRP Channels in the Lacrimal Gland

In lacrimal glands, some TRP channels are involved in tear secretion and inflammation. Among them, TRPM3 located in the apical membrane of lacrimal gland epithelium may promote the development of lacrimal gland in the early mouse embryo (Kanewska et al., 2020). TRPV1 is also expressed in lacrimal glands and may play a role in regulating Ca^2+^ and water transport (Martinez-Garcia et al., 2013). In addition, the role of TRPV4 in the lacrimal gland is the same as that in the salivary gland, which is also activated at moderate temperature and regulates the transporter ANO1 through the influx of Ca^2+^ to enhance the secretion of the lacrimal gland, which is manifested by the tearing effect of muscarinic (Derouiche et al., 2018). However, additional studies on the specific mechanisms of TRP channels in lacrimal glands remain to be conducted.

## Conclusion and Discussion

In this article we review the specific regulatory mechanisms of various TRP channels in some common glands (pancreas, salivary gland, lacrimal gland, adrenal gland, gallbladder, sweat gland). Except the tissues discussed above, TRP channels are also reported to have important functions in other tissues. TRPC5 together with TRPA1 are capable for cold sensing in teeth (Bernal et al., 2021). Meanwhile TRPC5, which is expressed in dopamine ARC neurons, has also been reported to regulate prolactin homeostasis (Blum et al., 2019). TRPM2, which is expressed in a subpopulation of neurons in hypothalamic, is a temperature sensor and able to regulates the fever response (Song et al., 2016).

As summarized above, TRP channels are been participated in pathology of diverse system. Therefore, drug discovery targeting TRP channels to cure different disorders has grown. Moreover, majority of TRP channels are located at the cell surface, which means they are easily accessible target. Many members of the TRP channel superfamily are abundantly expressed in the pancreas, and their activity is closely related to the physiological and pathological processes of the pancreas. TRPA1 can be activated by a variety of substances to promote Ca^2+^ influx to induce insulin secretion, but maintaining the activated state for a long time will instead inhibit insulin secretion (Steinritz et al., 2018). TRPV1 is expressed in sensory nerve fibers in the mouse pancreas and does not mediate insulin secretion (Diaz-Garcia et al., 2014), but is closely linked to the physiological function of β-cells (Razavi et al., 2006; Najder et al., 2018; Zhong et al., 2019), in addition to mediating the release of inflammatory mediators and the production of pain sensation in acute pancreatitis (Schwartz et al., 2011; Schwartz et al., 2013). There is no disease-modifying treatment for people with type 2 diabetes mellitus. Accumulating evidence suggests that pharmacological blockade of TRPV1 with the small molecule antagonist BCTC291 improves oral glucose tolerance and glucose-stimulated insulin secretion. Another TRPV1 antagonist, XEN-D0501, is currently undergoing phase II clinical trials in T2DM patients with good tolerability and safety. The combination of TRPV1 and TRPA1 antagonists also protects sensory nerves and prevents cognitive decline in patients with T2DM. TRPC1, TRPC3, and TRPC4 is expressed in pancreatic β-cells and can be activated by various pathways such as G-protein-coupled receptors and mechanical stimuli to mediate insulin secretion under physiological conditions (Hayes et al., 2013; Park et al., 2013; Srivastava et al., 2018), but the activation of these TRPCs in PDAC is a major cause of PSCs migration and chemotaxis, which will aggravate the development of the disease. These TRPCs have the potential to be a biological indicator for the diagnosis and treatment of PDAC, but there is no excessive literature to support the conjecture. The TRPM subfamily is the most reported TRP channel expressed in the pancreas, in which TRPM2, TRPM4, and TRPM5 mediates Ca^2+^ influx with significant differences in activation patterns, while TRPM3 and TRPM7 is also permeable to Zn^2+^, and they all play a role in insulin secretion. It is worth noting that TRPM2, TRPM7, and TRPM8 is involved in the progression of pancreatic cancer, and TRPM7 and TRPM8 is overexpressed in pancreatic cancer tissues and has become a marker for judging cancer cell proliferation in clinical practice (Yee et al., 2011; Yee et al., 2012a). Interestingly, they play an opposite role in pancreatic cancer. TRPM7 has a protective effect on pancreatic cancer, but TRPM8 can promote the progression of the disease, which provides a new target and idea for the treatment of pancreatic cancer. In addition, other TRPMs channels have not been reported in pancreatic diseases, and their role in the disease is still unknown. Many TRPC members are expressed in the mammary gland and are closely associated with the development and metastasis of breast cancer. Adriamycin is one of the drugs for the treatment of breast cancer in clinical practice, but the up-regulation of TRPC5 makes tumor cells resistant to adriamycin and promotes the metastasis of tumor cells (Ma et al., 2012b; Zhang et al., 2017). Blocking TRPC5 activation may provide new clinical treatment options for adriamycin-resistant breast cancer. In addition, digoxin may have a therapeutic effect on Triple-negative breast cancers (TNBC) cell lines with high TRPC1 and TRPC4 expression (Grant et al., 2019). Some other diseases and related drugs currently studied are summarized in Supplementary Table S1.

Much of our understanding of the contribution of TRP channels to disease comes from studies of *in vitro* systems or preclinical rodent models, which do not always reflect human disease. In this context, endocrine-related diseases caused by abnormal TRP channels provide some uncertainty. In terms of drug discovery, diseases caused by functional overactivation of TRP channels may be easier to treat because small molecules (e.g., targeted nanodrugs) can inhibit overactivation of TRP channels on the surface of cell membranes; Diseases caused by loss of TRP channel function, especially gene mutations, are difficult to target with small molecules and may require a less-validated approach, such as gene therapy, DNA-based nanostructures, or organoid therapy, to restore normal TRP channel function. In addition to directly reversing dysfunctional TRP channels, there may be benefits in regulating the function of intact TRP channels through therapeutic interventions when diseases associated with these channel diseases are caused by other genetic mutations or environmental factors.

In summary, due to the diversity and specificity of TRP channels, a variety of customized TRP channel-based structures have been created. The mechanism of action of endocrine-related TRP channels summarized in this paper provides valuable directions for biomedical development. On salivary secretion, the role of TRPC1, TRPM2 and TRPV4 in the physiological and pathological processes of salivary glands has been to be explored (Ambudkar, 2016; Liu et al., 2018). Some TRP channel superfamily members are expressed in the lacrimal gland and mainly mediate the development and secretion of the lacrimal gland, but the mechanism remains to be studied (Martinez-Garcia et al., 2013; Derouiche et al., 2018; Kanewska et al., 2020). TRPA1, TRPV1, TRPM4 and TRPM7 has been shown to affect adrenaline production (Iwasaki et al., 2008; Mathar et al., 2010b; Ferreira et al., 2019; Yeung et al., 2021). TRP channels are also expressed in sweat and lacrimal glands, and there is currently insufficient evidence for the association of TRP channels with sweat gland secretion, TRPV6 and TRPM3 promote cell development in sweat and lacrimal glands, respectively, and TRPV4 plays a role in enhancing lacrimal gland secretion (Derouiche et al., 2018; Lehen’kyi et al., 2007; Kanewska et al., 2020). TRPM7, TRPM8, and TRPV6 have been used as diagnostic and prognostic markers of breast cancer in clinical practice (Lehen’kyi et al., 2012; Van Haute et al., 2010; Chan et al., 1991; Adams et al., 2003; Zhuang et al., 2002; Tsavaler et al., 2001; Wang et al., 2009), but because the molecular markers are still insufficient in the judgment of prognosis, and it is necessary to explore the correlation between more types of TRP channels and breast cancer, in addition to the lack of specific drug inhibitors of these channels is also an obstacle that must be overcome.

These problems are real, but may not be insurmountable, and the potential benefits are considerable. If drug discovery companies can find creative ways to harness TRP pathways in diseases, they may be able to develop novel, state-of-the-art drugs.

## Abbreviations

AM, adrenal medulla; ADPR, ADP ribose; AITC, Allyl isothiocyanate; ANO1, Anoctamin 1; AQP5, Aquaporin 5; ASNA, adrenal sympathetic nerve activity; BK, Ca2+ -activated K+ channels; BAT, brown adipose tissue; cAMP, cyclic adenosine monophosphate; CAN, cinnamaldehyde; CCh, carbachol; DAG, diacyl glycerol; CGRP, calcitonin-gene-related peptide; CLICs, intracellular chloride channels; [Ca2+]i, cytosolic [Ca2+]; CCK, Cholecystokinin; ER, endoplasmic reticulum; GBC, gallbladder cancer; EMT, epithelial-to-mesenchymal transition; ET-1, endothelin 1; GPCR, G protein-coupled receptor; GBSM, gallbladder smooth muscle; GPR, G protein-coupled receptor; GLP-1, glucagon-like peptide 1; GRE, glucocorticoid response element; IDC, invasive ductal carcinoma; IP3, inositol 1,4,5-triphosphate; KCa, Ca2+-dependent K+; NAD+, Nicotinamide adenine dinucleotide; NKCC1, Na+/K+/2Cl− cotransporter 1; OA, oleuropein aglycone; NSC, nonselective cation channels; PACAP, pituitary adenylate cyclase-activating polypeptide; PDAC, pancreatic ductal adenocarcinoma; PDX-1, pancreatic and duodenal homeobox 1; PIP2, phosphatidylinositol bisphosphate; PregS, pregnenolone sulfate; pSS, Sjögren’s syndromeSP, substance P; STIM, stromal interaction molecule; SWD, sweetbread; socs3a, suppressor of cytokine signaling 3a; PKC, protein kinase C; PLC, phospholipase C; PSC, pancreatic stellate cell; ROS, reactive oxygen species; RVI, regulatory volume increase; RVD, regulatory volume decrease; SOC, store-operated Ca2+; SOCE, store-operated Ca2+ entry; TRP, transient receptor potential; TRPC, transient receptor potential canonical; TRPV, transient receptor potential vanilloid, TRPM, transient receptor potential melastatin, TRPA, transient receptor potential ankyrin; TRPP, transient receptor potential polycystic; TRPML, transient receptor potential mucolipin; Xbp1s, X-box binding proteins; UPR, unfolded protein response; ZFHX3, zinc finger homeobox 3; 4a-PDD, 4-a-phorbol12,13-didecanoat.
