# Supplementary material for: TRP Channels as Molecular Targets to Relieve Endocrine-Related Diseases
Source: Front Mol Biosci. 2022 Apr 28;9:895814. doi: 10.3389/fmolb.2022.895814 (PMC9095829; doi:10.3389/fmolb.2022.895814)
Supplement: Supplementary file 1 [file Table1.DOCX]

| TRP name | Gland | Agent | Drug | Pathway | Related disease | Refs. |
| --- | --- | --- | --- | --- | --- | --- |
| TRPA1 | Pancreas | 2-hydroxyestradiol ,  4-hydroxylated catechol estrogen,  Cinnamaldehyde,  allyl isothiocyanate (AITC),  hydrogen peroxide (H_2_O_2_),  4-hydroxynonenal (4-HNE),  Cyclopentenone prostaglandins (PGJ_2_),  methylglyoxal (MG),  HC030031,  AP-18 | Glibenclamide,  Methylglyoxal | collaborate with KATP channel blocking;  interfere with the transcription of PDX-1 | Affects cell cycle and cell migration in PDAC.  Promotes normal secretion of islets in diabetes.  Conduct pain in pancreatitis | (1-8) |
|  | Adrenal gland | Cinnamaldehyde,  allyl isothiocyanate,  β-eudesmol,  oleuropein aglycone |  |  |  | (9-11) |
| TRPC1 | Pancreas | Protein kinase C,  pressure | 2-aminoethoxydiphenyl borate (2-APB) | Mediate SOCE;  TRPC1 and Orai1 form cation channels that mediate calcium entry and are regulated by STIM1 via intermolecular electrostatic interaction. | In Type 2 Diabetes, SOCE is impaired and TRPC1 action is abnormal.  In PDAC, it promotes mechanical conduction, cell migration, and chemotactic neutrophils. | (12-17) |
|  | Salivary gland |  | Thapsigargin (Tg)，  SKF96365，  Febuxostat， |  |  | (18-20) |
|  | Adrenal gland | Muscarinic receptors |  | TRPC1 – TRPC4 heteromeric channels function as store-operated Ca^2+^ entry channels | Metabolic syndrome | (21, 22) |
|  | Mammary gland | Stromal interaction molecule 1 |  | TGF-β -induced EMT relies on Ca^2+^ entry through the TRPC1-STIM1 complex | Breast cancer | (23) |
| TRPC3 | Pancreas | GRP40,  phospholipase C,  PKC,  pyrazole-3 (Pyr3),  microRNA-26a (miR-26a) | Fasiglifam | Mediate SOCE | Upregulated in PDAC and cooperated with K_Ca_3.1 channels to promote PSCs migration.  A target for prevention of pancreatic damage in acute pancreatitis. | (24-28) |
|  | Salivary Gland |  | Pyr3 | Mediate SOCE;  TRPC3-TRPC1 and Orai1 share a common ion channel and are regulated by STIM1. | A target for prevention of Sjögren syndrome | (29, 30) |
|  | Adrenal gland | GPCRs,  RTKs,  diacylglycerol |  | Mediate ROCC |  | (31) |
|  | Mammary gland | Arachidonic acid,  linolenic acid,  polyunsaturated fatty acids,  diacylglycerol |  | Mediate SOCE;  LPA-LPAR3-TRPC3 pathway |  | (32, 33) |
| TRPC4 | Pancreas | Ca^2+^ store depletion,  protein histidine phosphatase 1 (PHPT-1),  Leptin | Dexamethasone | Mediate SOCE | Mutations may occur in the congenital hyperinsulinism (CHI) | (34-36) |
|  | Adrenal gland | Histamine 1R,  leptin |  | Leptin-JAK-IP3K pathway |  | (37, 38) |
|  | Mammary gland | Englerin A | Digoxin | TRPC4-mediated Ca^2+^ influx induces proliferation and metastasis of cancer cells | Triple-negative breast cancers | (39) |
| TRPC5 | Adrenal gland | GPCRs,  RTKs |  | Gq/11-PLCβ pathway or Ca^2+^ store depleting pathway | metabolic syndrome | (22) |
|  | Mammary gland | Englerin A,  T5E3,  lenti-TRPC5-DN,  2-APB | Adriamycin | CaMKKβ/AMPKα/mTOR pathway;  TRPC5–NFATc3–P-gp pathway | Triple-negative breast cancers | (40-42) |
| TRPC6 | Pancreas | Anthranilic acid (ACA)  hypoxic condition,  microRNA-26a (miR-26a) | N-phenylcinnamides | Mediate SOCE | In Pancreatic cancer, TRPC6 is activated by hypoxia, which promotes migration of PSCs.  Therapeutic targets for acute pancreatitis | (28, 43, 44) |
|  | Adrenal gland | GPCRs,  RTKs,  diacylglycerol,  IP3R/RyanodineR coupling |  | Gq/11-PLCβ pathway;  Ca^2+^ store depleting pathway | metabolic syndrome | (22, 23) |
|  | Mammary gland | diacylglycerol |  | G protein-coupled receptor signaling pathway | Breast cancer | (45-47) |
| TRPM2 | Pancreas | Adenine dinucleotides (ADP ribose, NAD^+^, 2′-deoxy-ADPR)  H_2_O_2_, ROS,  Ca^2+^,  anthranilic acid (ACA),  temperature variation,  arachidonic acid,  PKA,  Alloxan,  GLP-1,  Ghrelin, | Epinephrine,  N-phenylcinnamides,  flufenamic acid,  clotrimazole, econazole,  Imeglimin | Activated by existing Ca^2+^, mediates the influx of cations such as Na^+^, activates VGCC. | In PDAC, ROS is sensed to promote the survival of tumor cells.  Diet-induced obesity and insulin resistance are promoted.  In diabetes, it mediates cell death.  Pancreatic cancer is promoted by PKC/MAPK pathway.  A potential treatment option for biliary pancreatitis. | (44, 48-57) |
|  | Salivary Gland | ROS，  ADP ribose,  NAD^+^,  Osmotic pressure | Tempol,  3-aminobenzamide,  TPL (4-hydroxy-2,2,6,6-tetramethylpiperidine-N-oxyl) | Mediate SOCE | Involved in post-radiation inflammatory damage and pSS | (18, 58) |
| TRPM3 | Pancreas | Pregnenolone sulfate,  Progesterone,  3,4-dihydro-N-(5-methyl-3-isoxazolyl)-a-phenyl-1(2H)-quinolineacetamide (CIM0216),  phosphatidylinositol 4,5-biphosphate (PIP2),  heat | Mefenamic acid,  nifedipine (Nif),  clotrimazole | Mediate cation influx such as Na^+^ and Zn^2+^, and activate VGCC | In insulinoma cells, it is indirectly involved in inducing signaling cascade. | (59-65) |
| TRPM4 | Pancreas | Ca^2+^,  adenine nucleotides,  glibenclamide,  PKA and PKC,  PIP2,  GLP-1 | 9-phenanthrol | Activated by extracellular Ca^2+^, mediates the influx of cations such as Na^+^, blocks KATP channel, activates VGCC. |  | (48, 52, 66) |
|  | Adrenal gland | PACAP |  | PACAP activate TRPM4 in PKC-dependent manner | Hypertension | (67) |
|  | Mammary gland | 9-phenanthrol,  aryloxyacyl-anthranilic 5 |  | TRPM4 may induce EMT through the β -catenin signaling pathway | Breast cancer | (68-70) |
| TRPM5 | Pancreas | Ca^2+^,  arachidonic acid,  PIP2,  PKC  G-protein coupled heterodimeric sweet taste receptor T1R2/T1R3,  GLP-1,  L-arginine | Stevioside | Activated by extracellular Ca^2+^, mediates the influx of cations such as Na^+^, blocks KATP channel, activates VGCC. | Dysfunction contributes to type 2 diabetes,  A therapeutic target of stevioside in type 2 diabetes mellitus,  Down-regulated as a biomarker in type 1 diabetes. | (48, 52, 66, 71-73) |
| TRPM7 | Pancreas |  |  |  | In pancreatic stellate cells of PDAC, it promotes mechanical conduction and neutrophil chemotaxis.  Biomarker and target in pancreatic adenocarcinoma, | (52, 74, 75) |
|  | Adrenal gland |  |  | activated pSTAT3 epigenetically regulates the transcription of TRPM7 | Obesity,  hypertension | (76) |
|  | Mammary gland | Carvacrol,  ginsenoside Rd | Waixenicin A | [Ca^2+^]_i_ regulated by TRPM7 induce EMT and the cell cycle | Breast cancer | (77, 78) |
| TRPM8 | Pancreas | anthranilic acid (ACA),  menthol and icilin,  N-(3-aminopropyl)-2-{[(3-methylphenyl) methyl]oxy}-N-(2-thienylmethyl)benzamide hydrochloride salt | N-phenylcinnamides |  | Biomarker and target in pancreatic adenocarcinoma, | (44, 75) |
|  | Mammary gland | Menthol,  Frescolat ML,  Frescolat MAG,  Coolact P |  | Mediate VGCC | Breast cancer | (78) |
| TRPV1 | Pancreas | capsaicin |  |  | In type 1 diabetes, mediate insulin resistance and neurogenic inflammation,  Conduct pain in pancreatitis | (5, 8, 79, 80) |
|  | Adrenal gland | Capsaicin,  n-tert-butylcyclohexanol | Modafinil |  | chronic fatigue syndrome,  Neuropathic pain,  severe sepsis | (81-83) |
|  | Mammary gland | Capsaicin,  reactive oxygen species | Docetaxel,  tramadol |  | Breast cancer | (84, 85) |
| TRPV2 | Pancreas | heat,  mechanical stretch,  osmotic swelling,  phosphatidylinositol 3 kinase (PI3K),  anti-aging gene *Klotho* | Tranilast,  Nifedipine,  Diazoxide,  somatostatin | Translocation from the cytoplasm to the membrane | In cystic fibrosis, dysregulation of the TRPV2 signaling pathway leads to defective macrophage function, leading to chronic infection. | (26, 48, 86-88) |
|  | Mammary gland | LL-37 |  | PI3K/AKT-TRPV2 pathway | Breast cancer | (89) |
| TRPV3 | Mammary gland |  | Lapatinib |  | Breast cancer | (98) |
| TRPV4 | Pancreas | heat,  mechanical stretch,  osmotic swelling,  extracellular signal-regulated kinase 1/2（ERK1/2） | 4α-phorbol 12,13-didecanoate (4-αPDD),  GSK1016790A |  | In pancreatic stellate cells of PDAC, it promotes mechanical conduction and neutrophil chemotaxis.  Mediate pressure-induced pancreatitis.  Therapeutic targets for type 2 diabetes | (48, 52, 74, 90-93) |
|  | Salivary Gland | Heat,  Osmotic pressure,  4α-phorbol-12,13 didecanoate, | GSK1016790A |  |  | (18) |
|  | Mammary gland | 4a-PDD,  GSK1016790A,  body osmolarity,  mechanosensation,  temperature sensing |  | Activation of TRPV4 induces BK channel opening and claudin protein downregulation;  TRPV4 can deform cells through actin depolymerization and V-ASP phosphorylation | Lactation deficiency,  Breast cancer | (94, 95) |
|  | Sweat gland |  |  |  |  | (96) |
| TRPV5 | Pancreas | Estradiol |  |  |  | (97) |
|  | Mammary gland |  | Lapatinib |  | Breast cancer | (98) |
| TRPV6 | Pancreas | Estradiol |  |  | In pancreatic cancer, TRPV6 mediates cell proliferation, invasion and migration, and is a biomarker. | (99, 100) |
|  | Mammary gland | Diacylglycerol | Lapatinib,  tamoxifen | Ca^2+^ influx mediated by TRPV6 activation induces keratinocyte differentiation | Breast cancer | (101) |
| TRPP2 | Gallbladder | IP3R |  | PLC-IP3 pathway |  | (102) |

**REFERENCES**

1. Ma W, Chen X, Cerne R, Syed SK, Ficorilli JV, Cabrera O, et al. Catechol estrogens stimulate insulin secretion in pancreatic beta-cells via activation of the transient receptor potential A1 (TRPA1) channel. *J Biol Chem*. (2019) 294: 2935-46. doi: 10.1074/jbc.RA118.005504

2. Babes A, Fischer MJ, Filipovic M, Engel MA, Flonta ML, Reeh PW. The anti-diabetic drug glibenclamide is an agonist of the transient receptor potential Ankyrin 1 (TRPA1) ion channel. *Eur J Pharmacol*. (2013) 704: 15-22. doi: 10.1016/j.ejphar.2013.02.018

3. Anand P, Murali KY, Tandon V, Murthy PS, Chandra R. Insulinotropic effect of cinnamaldehyde on transcriptional regulation of pyruvate kinase, phosphoenolpyruvate carboxykinase, and GLUT4 translocation in experimental diabetic rats. *Chem Biol Interact*. (2010) 186: 72-81. doi: 10.1016/j.cbi.2010.03.044

4. Cao DS, Zhong L, Hsieh TH, Abooj M, Bishnoi M, Hughes L, et al. Expression of transient receptor potential ankyrin 1 (TRPA1) and its role in insulin release from rat pancreatic beta cells. *Plos One*. (2012) 7: e38005. doi: 10.1371/journal.pone.0038005

5. Steinritz D, Stenger B, Dietrich A, Gudermann T, Popp T. TRPs in tox: Involvement of transient receptor Potential-Channels in Chemical-Induced organ Toxicity-A structured review. *Cells-Basel*. (2018) 7. doi: 10.3390/cells7080098

6. Cojocaru F, Selescu T, Domocos D, Marutescu L, Chiritoiu G, Chelaru NR, et al. Publisher Correction: Functional expression of the transient receptor potential ankyrin type 1 channel in pancreatic adenocarcinoma cells. *Sci Rep*. (2021) 11: 8853. doi: 10.1038/s41598-021-88169-9

7. Andersson DA, Gentry C, Light E, Vastani N, Vallortigara J, Bierhaus A, et al. Methylglyoxal evokes pain by stimulating TRPA1. *Plos One*. (2013) 8: e77986. doi: 10.1371/journal.pone.0077986

8. Schwartz ES, La JH, Scheff NN, Davis BM, Albers KM, Gebhart GF. TRPV1 and TRPA1 antagonists prevent the transition of acute to chronic inflammation and pain in chronic pancreatitis. *J Neurosci*. (2013) 33: 5603-11. doi: 10.1523/JNEUROSCI.1806-12.2013

9. Iwasaki Y, Tanabe M, Kobata K, Watanabe T. TRPA1 agonists--allyl isothiocyanate and cinnamaldehyde--induce adrenaline secretion. *Biosci Biotechnol Biochem*. (2008) 72: 2608-14. doi: 10.1271/bbb.80289

10. Ohara K, Katayama M, Nagai K. Β-eudesmol, an oxygenized sesquiterpene, affects efferent adrenal sympathetic nerve activity via transient receptor potential ankyrin 1 in rats. *Neurosci Lett*. (2018) 684: 18-24. doi: 10.1016/j.neulet.2018.06.057

11. Oi-Kano Y, Iwasaki Y, Nakamura T, Watanabe T, Goto T, Kawada T, et al. Oleuropein aglycone enhances UCP1 expression in brown adipose tissue in high-fat-diet-induced obese rats by activating β-adrenergic signaling. *The Journal of Nutritional Biochemistry*. (2017) 40: 209-18. doi: 10.1016/j.jnutbio.2016.11.009

12. Dyachok O, Gylfe E. Store-operated influx of Ca(2+) in pancreatic beta-cells exhibits graded dependence on the filling of the endoplasmic reticulum. *J Cell Sci*. (2001) 114: 2179-86. doi: 10.1242/jcs.114.11.2179

13. Xu J, Zhang W, Cui W, Shi B, Wang H. PKCalpha promotes insulin secretion via TRPC1 phosphorylation in INS-1E cells. *Biosci Biotechnol Biochem*. (2019) 83: 1676-82. doi: 10.1080/09168451.2019.1617106

14. Zeng W, Yuan JP, Kim MS, Choi YJ, Huang GN, Worley PF, et al. STIM1 gates TRPC channels, but not Orai1, by electrostatic interaction. *Mol Cell*. (2008) 32: 439-48. doi: 10.1016/j.molcel.2008.09.020

15. Kono T, Tong X, Taleb S, Bone RN, Iida H, Lee CC, et al. Impaired Store-Operated calcium entry and STIM1 loss lead to reduced insulin secretion and increased endoplasmic reticulum stress in the diabetic beta-Cell. *Diabetes*. (2018) 67: 2293-304. doi: 10.2337/db17-1351

16. Lindemann O, Strodthoff C, Horstmann M, Nielsen N, Jung F, Schimmelpfennig S, et al. TRPC1 regulates fMLP-stimulated migration and chemotaxis of neutrophil granulocytes. *Biochim Biophys Acta*. (2015) 1853: 2122-30. doi: 10.1016/j.bbamcr.2014.12.037

17. Dong H, Shim KN, Li JM, Estrema C, Ornelas TA, Nguyen F, et al. Molecular mechanisms underlying Ca2+-mediated motility of human pancreatic duct cells. *Am J Physiol Cell Physiol*. (2010) 299: C1493-503. doi: 10.1152/ajpcell.00242.2010

18. Liu X, Ong HL, Ambudkar I. TRP channel involvement in salivary Glands-Some good, some bad. *Cells-Basel*. (2018) 7. doi: 10.3390/cells7070074

19. Sun Y, Birnbaumer L, Singh BB. TRPC1 regulates calcium-activated chloride channels in salivary gland cells. *J Cell Physiol*. (2015) 230: 2848-56. doi: 10.1002/jcp.25017

20. Abdelzaher WY, Nassan MA, Ahmed SM, Welson NN, El-Saber BG, Khalaf HM. Xanthine oxidase inhibitor, febuxostat is effective against 5-Fluorouracil-Induced parotid salivary gland injury in rats via inhibition of oxidative stress, inflammation and targeting TRPC1/CHOP signalling pathway. *Pharmaceuticals (Basel)*. (2022) 15. doi: 10.3390/ph15020232

21. Harada K, Matsuoka H, Inoue M. STIM1-dependent membrane insertion of heteromeric TRPC1/4 channels in response to muscarinic receptor stimulation. *J Cell Sci*. (2019). doi: 10.1242/jcs.227389

22. Hu G, Oboukhova EA, Kumar S, Sturek M, Obukhov AG. Canonical transient receptor potential channels expression is elevated in a porcine model of metabolic syndrome. *Mol Endocrinol*. (2009) 23: 689-99. doi: 10.1210/me.2008-0350

23. Schaar A, Sukumaran P, Sun Y, Dhasarathy A, Singh BB. TRPC1-STIM1 activation modulates transforming growth factor beta-induced epithelial-to-mesenchymal transition. *Oncotarget*. (2016) 7: 80554-67. doi: 10.18632/oncotarget.12895

24. Storck H, Hild B, Schimmelpfennig S, Sargin S, Nielsen N, Zaccagnino A, et al. Ion channels in control of pancreatic stellate cell migration. *Oncotarget*. (2017) 8: 769-84. doi: 10.18632/oncotarget.13647

25. Yamada H, Yoshida M, Ito K, Dezaki K, Yada T, Ishikawa SE, et al. Potentiation of glucose-stimulated insulin secretion by the GPR40-PLC-TRPC pathway in pancreatic beta-Cells. *Sci Rep*. (2016) 6: 25912. doi: 10.1038/srep25912

26. Islam MS. Molecular regulations and functions of the transient receptor potential channels of the islets of langerhans and insulinoma cells. *Cells-Basel*. (2020) 9. doi: 10.3390/cells9030685

27. Kim MS, Hong JH, Li Q, Shin DM, Abramowitz J, Birnbaumer L, et al. Deletion of TRPC3 in mice reduces store-operated Ca2+ influx and the severity of acute pancreatitis. *Gastroenterology*. (2009) 137: 1509-17. doi: 10.1053/j.gastro.2009.07.042

28. Du W, Liu G, Shi N, Tang D, Ferdek PE, Jakubowska MA, et al. A microRNA checkpoint for Ca(2+) signaling and overload in acute pancreatitis. *Mol Ther*. (2022). doi: 10.1016/j.ymthe.2022.01.033

29. Ambudkar IS. Calcium signalling in salivary gland physiology and dysfunction. *J Physiol*. (2016) 594: 2813-24. doi: 10.1113/JP271143

30. Kim MS, Lee KP, Yang D, Shin DM, Abramowitz J, Kiyonaka S, et al. Genetic and pharmacologic inhibition of the Ca2+ influx channel TRPC3 protects secretory epithelia from Ca2+-dependent toxicity. *Gastroenterology*. (2011) 140: 2107-15, 2111-5. doi: 10.1053/j.gastro.2011.02.052

31. Obukhov AG, Nowycky MC. TRPC4 can be activated by g-protein-coupled receptors and provides sufficient ca2+ to trigger exocytosis in neuroendocrine cells. *J Biol Chem*. (2002) 277: 16172-8. doi: 10.1074/jbc.M111664200

32. Zhang H, Zhou L, Shi W, Song N, Yu K, Gu Y. A mechanism underlying the effects of polyunsaturated fatty acids on breast cancer. *Int J Mol Med*. (2012) 30: 487-94. doi: 10.3892/ijmm.2012.1022

33. Hirata N, Yamada S, Yanagida S, Ono A, Yasuhiko Y, Nishida M, et al. Lysophosphatidic acid promotes the expansion of cancer stem cells via TRPC3 channels in Triple-Negative breast cancer. *Int J Mol Sci*. (2022) 23: 1967. doi: 10.3390/ijms23041967

34. Srivastava S, Li Z, Soomro I, Sun Y, Wang J, Bao L, et al. Regulation of KATP channel trafficking in pancreatic beta-Cells by protein histidine phosphorylation. *Diabetes*. (2018) 67: 849-60. doi: 10.2337/db17-1433

35. Park SH, Ryu SY, Yu WJ, Han YE, Ji YS, Oh K, et al. Leptin promotes K(ATP) channel trafficking by AMPK signaling in pancreatic beta-cells. *Proc Natl Acad Sci U S A*. (2013) 110: 12673-8. doi: 10.1073/pnas.1216351110

36. Wie J, Kim J, Ha K, Zhang YH, Jeon JH, So I. Dexamethasone activates transient receptor potential canonical 4 (TRPC4) channels via Rasd1 small GTPase pathway. *Pflugers Arch*. (2015) 467: 2081-91. doi: 10.1007/s00424-014-1666-0

37. Tesfai Y, Brereton HM, Barritt GJ. A diacylglycerol-activated Ca2+ channel in PC12 cells (an adrenal chromaffin cell line) correlates with expression of the TRP-6 (transient receptor potential) protein. *Biochem J*. (2001) 358: 717-26. doi: 10.1042/0264-6021:3580717

38. Gavello D, Carbone E, Carabelli V. Leptin-mediated ion channel regulation: PI3K pathways, physiological role, and therapeutic potential. *Channels*. (2016) 10: 282-96. doi: 10.1080/19336950.2016.1164373

39. Grant CV, Carver CM, Hastings SD, Ramachandran K, Muniswamy M, Risinger AL, et al. Triple-negative breast cancer cell line sensitivity to englerin a identifies a new, targetable subtype. *Breast Cancer Res Tr*. (2019) 177: 345-55. doi: 10.1007/s10549-019-05324-7

40. Wang T, Ning K, Lu TX, Sun X, Jin L, Qi X, et al. Increasing circulating exosomes-carrying TRPC5 predicts chemoresistance in metastatic breast cancer patients. *Cancer Sci*. (2017) 108: 448-54. doi: 10.1111/cas.13150

41. Ma X, Cai Y, He D, Zou C, Zhang P, Lo CY, et al. Transient receptor potential channel TRPC5 is essential for P-glycoprotein induction in drug-resistant cancer cells. *Proceedings of the National Academy of Sciences*. (2012) 109: 16282-7. doi: 10.1073/pnas.1202989109

42. Zhang P, Liu X, Li H, Chen Z, Yao X, Jin J, et al. TRPC5-induced autophagy promotes drug resistance in breast carcinoma via CaMKKβ/AMPKα/mTOR pathway. *Sci Rep-Uk*. (2017) 7. doi: 10.1038/s41598-017-03230-w

43. Nielsen N, Kondratska K, Ruck T, Hild B, Kovalenko I, Schimmelpfennig S, et al. TRPC6 channels modulate the response of pancreatic stellate cells to hypoxia. *Pflugers Arch*. (2017) 469: 1567-77. doi: 10.1007/s00424-017-2057-0

44. Harteneck C, Frenzel H, Kraft R. N-(p-amylcinnamoyl)anthranilic acid (ACA): A phospholipase A(2) inhibitor and TRP channel blocker. *Cardiovasc Drug Rev*. (2007) 25: 61-75. doi: 10.1111/j.1527-3466.2007.00005.x

45. Soboloff J, Spassova M, Xu W, He L, Cuesta N, Gill DL. Role of endogenous TRPC6 channels in Ca2+ signal generation in A7r5 smooth muscle cells. *The Journal of biological chemistry*. (2005) 280: 39786-94.

46. Trebak M, Vazquez G, Bird GSJ, Putney JWJ. The TRPC3/6/7 subfamily of cation channels. *Cell Calcium*. (2003) 33: 451-61.

47. Guilbert A, Dhennin-Duthille I, Hiani YE, Haren N, Khorsi H, Sevestre H, et al. Expression of TRPC6 channels in human epithelial breast cancer cells. *Bmc Cancer*. (2008) 8: 125. doi: 10.1186/1471-2407-8-125

48. Islam MS. TRP channels of islets. *Adv Exp Med Biol*. (2011) 704: 811-30. doi: 10.1007/978-94-007-0265-3_42

49. Du J, Xie J, Yue L. Intracellular calcium activates TRPM2 and its alternative spliced isoforms. *Proc Natl Acad Sci U S A*. (2009) 106: 7239-44. doi: 10.1073/pnas.0811725106

50. McHugh D, Flemming R, Xu SZ, Perraud AL, Beech DJ. Critical intracellular Ca2+ dependence of transient receptor potential melastatin 2 (TRPM2) cation channel activation. *J Biol Chem*. (2003) 278: 11002-6. doi: 10.1074/jbc.M210810200

51. Csanady L, Torocsik B. Four Ca2+ ions activate TRPM2 channels by binding in deep crevices near the pore but intracellularly of the gate. *J Gen Physiol*. (2009) 133: 189-203. doi: 10.1085/jgp.200810109

52. Najder K, Musset B, Lindemann O, Bulk E, Schwab A, Fels B. The function of TRP channels in neutrophil granulocytes. *Pflugers Arch*. (2018) 470: 1017-33. doi: 10.1007/s00424-018-2146-8

53. Fliegert R, Bauche A, Wolf PA, Watt JM, Rozewitz MD, Winzer R, et al. 2'-Deoxyadenosine 5'-diphosphoribose is an endogenous TRPM2 superagonist. *Nat Chem Biol*. (2017) 13: 1036-44. doi: 10.1038/nchembio.2415

54. Eisfeld J, Luckhoff A. Trpm2. *Handb Exp Pharmacol*. (2007): 237-52. doi: 10.1007/978-3-540-34891-7_14

55. Funazaki S, Yoshida M, Yamada H, Kakei M, Kawakami M, Nagashima S, et al. A novel mechanism of imeglimin-mediated insulin secretion via the cADPR-TRP channel pathway. *J Diabetes Investig*. (2022) 13: 34-41. doi: 10.1111/jdi.13669

56. Lin R, Bao X, Wang H, Zhu S, Liu Z, Chen Q, et al. TRPM2 promotes pancreatic cancer by PKC/MAPK pathway. *Cell Death Dis*. (2021) 12: 585. doi: 10.1038/s41419-021-03856-9

57. Fanczal J, Pallagi P, Gorog M, Diszhazi G, Almassy J, Madacsy T, et al. TRPM2-mediated extracellular Ca(2+) entry promotes acinar cell necrosis in biliary acute pancreatitis. *J Physiol*. (2020) 598: 1253-70. doi: 10.1113/JP279047

58. Liu X, Cotrim A, Teos L, Zheng C, Swaim W, Mitchell J, et al. Loss of TRPM2 function protects against irradiation-induced salivary gland dysfunction. *Nat Commun*. (2013) 4: 1515. doi: 10.1038/ncomms2526

59. Majeed Y, Agarwal AK, Naylor J, Seymour VA, Jiang S, Muraki K, et al. Cis-isomerism and other chemical requirements of steroidal agonists and partial agonists acting at TRPM3 channels. *Br J Pharmacol*. (2010) 161: 430-41. doi: 10.1111/j.1476-5381.2010.00892.x

60. Majeed Y, Tumova S, Green BL, Seymour VA, Woods DM, Agarwal AK, et al. Pregnenolone sulphate-independent inhibition of TRPM3 channels by progesterone. *Cell Calcium*. (2012) 51: 1-11. doi: 10.1016/j.ceca.2011.09.005

61. Colsoul B, Vennekens R, Nilius B. Transient receptor potential cation channels in pancreatic beta cells. *Rev Physiol Biochem Pharmacol*. (2011) 161: 87-110. doi: 10.1007/112_2011_2

62. Held K, Kichko T, De Clercq K, Klaassen H, Van Bree R, Vanherck JC, et al. Activation of TRPM3 by a potent synthetic ligand reveals a role in peptide release. *Proc Natl Acad Sci U S A*. (2015) 112: E1363-72. doi: 10.1073/pnas.1419845112

63. Badheka D, Borbiro I, Rohacs T. Transient receptor potential melastatin 3 is a phosphoinositide-dependent ion channel. *J Gen Physiol*. (2015) 146: 65-77. doi: 10.1085/jgp.201411336

64. Uchida K, Demirkhanyan L, Asuthkar S, Cohen A, Tominaga M, Zakharian E. Stimulation-dependent gating of TRPM3 channel in planar lipid bilayers. *Faseb J*. (2016) 30: 1306-16. doi: 10.1096/fj.15-281576

65. Mayer SI, Muller I, Mannebach S, Endo T, Thiel G. Signal transduction of pregnenolone sulfate in insulinoma cells: Activation of Egr-1 expression involving TRPM3, voltage-gated calcium channels, ERK, and ternary complex factors. *J Biol Chem*. (2011) 286: 10084-96. doi: 10.1074/jbc.M110.202697

66. Shigeto M, Ramracheya R, Tarasov AI, Cha CY, Chibalina MV, Hastoy B, et al. GLP-1 stimulates insulin secretion by PKC-dependent TRPM4 and TRPM5 activation. *J Clin Invest*. (2015) 125: 4714-28. doi: 10.1172/JCI81975

67. Inoue M, Harada K, Matsuoka H. Mechanisms for pituitary adenylate cyclase-activating polypeptide-induced increase in excitability in guinea-pig and mouse adrenal medullary cells. *Eur J Pharmacol*. (2020) 872: 172956. doi: 10.1016/j.ejphar.2020.172956

68. Grand T, Demion M, Norez C, Mettey Y, Launay P, Becq F, et al. 9-Phenanthrol inhibits human TRPM4 but not TRPM5 cationic channels. *Brit J Pharmacol*. (2008) 153: 1697-705. doi: 10.1038/bjp.2008.38

69. Ozhathil LC, Delalande C, Bianchi B, Nemeth G, Kappel S, Thomet U, et al. Identification of potent and selective small molecule inhibitors of the cation channel TRPM4. *Brit J Pharmacol*. (2018) 175: 2504-19. doi: 10.1111/bph.14220

70. Wong KK, Hussain FA. TRPM4 is overexpressed in breast cancer associated with estrogen response and epithelial-mesenchymal transition gene sets. *Plos One*. (2020) 15: e233884. doi: 10.1371/journal.pone.0233884

71. Brixel LR, Monteilh-Zoller MK, Ingenbrandt CS, Fleig A, Penner R, Enklaar T, et al. TRPM5 regulates glucose-stimulated insulin secretion. *Pflugers Arch*. (2010) 460: 69-76. doi: 10.1007/s00424-010-0835-z

72. Krishnan K, Ma Z, Bjorklund A, Islam MS. Role of transient receptor potential melastatin-like subtype 5 channel in insulin secretion from rat beta-cells. *Pancreas*. (2014) 43: 597-604. doi: 10.1097/MPA.0000000000000027

73. Yip L, Fuhlbrigge R, Alkhataybeh R, Fathman CG. Gene expression analysis of the Pre-Diabetic pancreas to identify pathogenic mechanisms and biomarkers of type 1 diabetes. *Front Endocrinol (Lausanne)*. (2020) 11: 609271. doi: 10.3389/fendo.2020.609271

74. Fels B, Nielsen N, Schwab A. Role of TRPC1 channels in pressure-mediated activation of murine pancreatic stellate cells. *Eur Biophys J*. (2016) 45: 657-70. doi: 10.1007/s00249-016-1176-4

75. Yee NS, Chan AS, Yee JD, Yee RK. TRPM7 and TRPM8 ion channels in pancreatic adenocarcinoma: Potential roles as cancer biomarkers and targets. *Scientifica (Cairo)*. (2012) 2012: 415158. doi: 10.6064/2012/415158

76. Yeung BH, Griffiths K, Berger L, Paudel O, Shin M, Rui L, et al. Leptin induces epigenetic regulation of transient receptor potential melastatin 7 in rat adrenal pheochromocytoma cells. *Am J Resp Cell Mol*. (2021) 65: 214-21. doi: 10.1165/rcmb.2020-0374OC

77. Li L, He L, Wu Y, Zhang Y. Carvacrol affects breast cancer cells through TRPM7 mediated cell cycle regulation. *Life Sci*. (2021) 266: 118894. doi: 10.1016/j.lfs.2020.118894

78. Liu Z, Wu H, Wei Z, Wang X, Shen P, Wang S, et al. TRPM8: A potential target for cancer treatment. *J Cancer Res Clin*. (2016) 142: 1871-81. doi: 10.1007/s00432-015-2112-1

79. Razavi R, Chan Y, Afifiyan FN, Liu XJ, Wan X, Yantha J, et al. TRPV1+ sensory neurons control beta cell stress and islet inflammation in autoimmune diabetes. *Cell*. (2006) 127: 1123-35. doi: 10.1016/j.cell.2006.10.038

80. Suri A, Szallasi A. The emerging role of TRPV1 in diabetes and obesity. *Trends Pharmacol Sci*. (2008) 29: 29-36. doi: 10.1016/j.tips.2007.10.016

81. Ferreira LGB, Prevatto JP, Freitas HR, Reis RAM, Silva PMR, Martins MA, et al. Capsaicin inhibits lipopolysaccharide-induced adrenal steroidogenesis by raising intracellular calcium levels. *Endocrine*. (2019) 64: 169-75. doi: 10.1007/s12020-019-01849-5

82. Sarvaiya K, Goswami S. Investigation of the effects of vanilloids in chronic fatigue syndrome. *Brain Res Bull*. (2016) 127: 187-94. doi: 10.1016/j.brainresbull.2016.09.015

83. Arribas-Blázquez M, Olivos-Oré LA, Barahona MV, Sánchez De La Muela M, Solar V, Jiménez E, et al. Overexpression of P2X3 and P2X7 receptors and TRPV1 channels in adrenomedullary chromaffin cells in a rat model of neuropathic pain. *Int J Mol Sci*. (2019) 20: 155. doi: 10.3390/ijms20010155

84. Ertilav K, Nazıroğlu M, Ataizi ZS, Yıldızhan K. Melatonin and selenium suppress Docetaxel-Induced TRPV1 activation, neuropathic pain and oxidative neurotoxicity in mice. *Biol Trace Elem Res*. (2021) 199: 1469-87. doi: 10.1007/s12011-020-02250-4

85. Kim MH, Lee J, Kim K, Jun JH, Hwang HJ, Lee W, et al. Identification for antitumor effects of tramadol in a xenograft mouse model using orthotopic breast cancer cells. *Sci Rep-Uk*. (2021) 11. doi: 10.1038/s41598-021-01701-9

86. Hisanaga E, Nagasawa M, Ueki K, Kulkarni RN, Mori M, Kojima I. Regulation of calcium-permeable TRPV2 channel by insulin in pancreatic beta-cells. *Diabetes*. (2009) 58: 174-84. doi: 10.2337/db08-0862

87. Lin Y, Sun Z. Antiaging gene Klotho enhances glucose-induced insulin secretion by up-regulating plasma membrane levels of TRPV2 in MIN6 beta-cells. *Endocrinology*. (2012) 153: 3029-39. doi: 10.1210/en.2012-1091

88. Leveque M, Penna A, Le Trionnaire S, Belleguic C, Desrues B, Brinchault G, et al. Phagocytosis depends on TRPV2-mediated calcium influx and requires TRPV2 in lipids rafts: Alteration in macrophages from patients with cystic fibrosis. *Sci Rep*. (2018) 8: 4310. doi: 10.1038/s41598-018-22558-5

89. Siveen KS, Nizamuddin PB, Uddin S, Al-Thani M, Frenneaux MP, Janahi IA, et al. TRPV2: A cancer biomarker and potential therapeutic target. *Dis Markers*. (2020) 2020: 1-10. doi: 10.1155/2020/8892312

90. Skrzypski M, Kakkassery M, Mergler S, Grotzinger C, Khajavi N, Sassek M, et al. Activation of TRPV4 channel in pancreatic INS-1E beta cells enhances glucose-stimulated insulin secretion via calcium-dependent mechanisms. *Febs Lett*. (2013) 587: 3281-7. doi: 10.1016/j.febslet.2013.08.025

91. Billert M, Skrzypski M, Sassek M, Szczepankiewicz D, Wojciechowicz T, Mergler S, et al. TRPV4 regulates insulin mRNA expression and INS-1E cell death via ERK1/2 and NO-dependent mechanisms. *Cell Signal*. (2017) 35: 242-9. doi: 10.1016/j.cellsig.2017.03.018

92. Swain SM, Romac JM, Shahid RA, Pandol SJ, Liedtke W, Vigna SR, et al. TRPV4 channel opening mediates pressure-induced pancreatitis initiated by Piezo1 activation. *J Clin Invest*. (2020) 130: 2527-41. doi: 10.1172/JCI134111

93. Hu W, Ding Y, Li Q, Shi R, He Y. Transient receptor potential vanilloid 4 channels as therapeutic targets in diabetes and diabetes-related complications. *J Diabetes Investig*. (2020) 11: 757-69. doi: 10.1111/jdi.13244

94. Lee WH, Choong LY, Mon NN, Lu S, Lin Q, Pang B, et al. TRPV4 regulates breast cancer cell extravasation, stiffness and actin cortex. *Sci Rep-Uk*. (2016) 6. doi: 10.1038/srep27903

95. Islam MA, Mizusawa M, Sharmin MM, Hayashi S, Yonekura S. TRPV4 increases the expression of tight junction Protein-Encoding genes via XBP1 in mammary epithelial cells. *Animals*. (2020) 10: 1174. doi: 10.3390/ani10071174

96. Fujii N, Kenny GP, Amano T, Honda Y, Kondo N, Nishiyasu T. Evidence for TRPV4 channel induced skin vasodilatation through NOS, COX, and KCa channel mechanisms with no effect on sweat rate in humans. *Eur J Pharmacol*. (2019) 858: 172462. doi: 10.1016/j.ejphar.2019.172462

97. Irnaten M, Blanchard-Gutton N, Praetorius J, Harvey BJ. Rapid effects of 17beta-estradiol on TRPV5 epithelial Ca2+ channels in rat renal cells. *Steroids*. (2009) 74: 642-9. doi: 10.1016/j.steroids.2009.02.002

98. Wang X, Li G, Zhang Y, Li L, Qiu L, Qian Z, et al. Pan-Cancer analysis reveals genomic and clinical characteristics of TRPV Channel-Related genes. *Front Oncol*. (2022) 12. doi: 10.3389/fonc.2022.813100

99. Irnaten M, Blanchard-Gutton N, Harvey BJ. Rapid effects of 17beta-estradiol on epithelial TRPV6 Ca2+ channel in human T84 colonic cells. *Cell Calcium*. (2008) 44: 441-52. doi: 10.1016/j.ceca.2008.02.007

100. Song H, Dong M, Zhou J, Sheng W, Li X, Gao W. Expression and prognostic significance of TRPV6 in the development and progression of pancreatic cancer. *Oncol Rep*. (2018) 39: 1432-40. doi: 10.3892/or.2018.6216

101. Bolanz KA, Hediger MA, Landowski CP. The role of TRPV6 in breast carcinogenesis. *Mol Cancer Ther*. (2008) 7: 271-9. doi: 10.1158/1535-7163.MCT-07-0478

102. Zhong X, Fu J, Song K, Xue N, Gong R, Sun D, et al. The role of TRPP2 in agonist-induced gallbladder smooth muscle contraction. *Science China Life Sciences*. (2016) 59: 409-16. doi: 10.1007/s11427-015-4958-5

**校对报告**

当前使用的样式是 [Frontiers in Medicine]

当前文档包含的题录共124条

有0条题录存在必填字段内容缺失的问题

所有题录的数据正常
